# Host insulin stimulates *Echinococcus multilocularis* insulin signalling pathways and larval development

**DOI:** 10.1186/1741-7007-12-5

**Published:** 2014-01-27

**Authors:** Sarah Hemer, Christian Konrad, Markus Spiliotis, Uriel Koziol, Dominik Schaack, Sabine Förster, Verena Gelmedin, Britta Stadelmann, Thomas Dandekar, Andrew Hemphill, Klaus Brehm

**Affiliations:** 1 University of Würzburg, Institute of Hygiene and Microbiology, Josef-Schneider-Strasse 2, D-97080 Würzburg, Germany; 2 Department of Bioinformatics, University of Würzburg, Biocenter am Hubland, D-97074 Würzburg, Germany; 3 Institute of Parasitology, Vetsuisse Faculty, University of Berne, Länggass-Strasse 122, CH-3012 Bern, Switzerland

**Keywords:** Cestode, Tapeworm, *Echinococcus*, Echinococcosis, Insulin, Receptor kinase, Kinase inhibitor, Host-parasite interaction

## Abstract

**Background:**

The metacestode of the tapeworm *Echinococcus multilocularis* is the causative agent of alveolar echinococcosis, a lethal zoonosis. Infections are initiated through establishment of parasite larvae within the intermediate host’s liver, where high concentrations of insulin are present, followed by tumour-like growth of the metacestode in host organs. The molecular mechanisms determining the organ tropism of *E. multilocularis* or the influences of host hormones on parasite proliferation are poorly understood.

**Results:**

Using *in vitro* cultivation systems for parasite larvae we show that physiological concentrations (10 nM) of human insulin significantly stimulate the formation of metacestode larvae from parasite stem cells and promote asexual growth of the metacestode. Addition of human insulin to parasite larvae led to increased glucose uptake and enhanced phosphorylation of *Echinococcus* insulin signalling components, including an insulin receptor-like kinase, EmIR1, for which we demonstrate predominant expression in the parasite’s glycogen storage cells. We also characterized a second insulin receptor family member, EmIR2, and demonstrated interaction of its ligand binding domain with human insulin in the yeast two-hybrid system. Addition of an insulin receptor inhibitor resulted in metacestode killing, prevented metacestode development from parasite stem cells, and impaired the activation of insulin signalling pathways through host insulin.

**Conclusions:**

Our data indicate that host insulin acts as a stimulant for parasite development within the host liver and that *E. multilocularis* senses the host hormone through an evolutionarily conserved insulin signalling pathway. Hormonal host-parasite cross-communication, facilitated by the relatively close phylogenetic relationship between *E. multilocularis* and its mammalian hosts, thus appears to be important in the pathology of alveolar echinococcosis. This contributes to a closer understanding of organ tropism and parasite persistence in larval cestode infections. Furthermore, our data show that *Echinococcus* insulin signalling pathways are promising targets for the development of novel drugs.

## Background

The metacestode stage of the fox-tapeworm *Echinococcus multilocularis* is the causative agent of alveolar echinococcosis (AE), one of the most serious parasitic diseases in the Northern Hemisphere [1]. Initial infection of the intermediate host (rodents, humans) occurs through oral uptake of infectious eggs that contain the oncosphere. Upon hatching from the egg within the intermediate host’s intestine, the oncosphere penetrates the intestinal wall and gains access to the inner organs. Almost exclusively within the liver, the oncosphere then undergoes a metamorphic transition towards the metacestode that is driven by parasite stem cells [2]. Once formed as small cystic structures, the metacestode tissue proliferates and infiltrates host tissue like a malignant tumour, eventually giving rise to numerous protoscoleces that either develop into the strobilar adult stage, when transmitted to the definitive host, or ‘re-differentiate’ towards the metacestode, when distributed in the intermediate host see Additional file 1[1-3].

All larval developmental transitions of *E. multilocularis* as well as proliferation of metacestode tissue take place in close contact with the intermediate host’s endocrine and paracrine systems, which involve numerous evolutionarily conserved hormones, such as insulin or cytokines of the epidermal growth factor (EGF) and the transforming growth factor-β (TGF-β) families. Since the parasite expresses respective surface receptor kinases it has already been suggested that the host-parasite interplay in AE might rely on hormonal host-parasite cross communication [2], although little information on the underlying interaction mechanisms is currently available. Of particular interest in the case of *E. multilocularis* are possible effects of host-derived insulin since, in mammalian hosts, the highest concentrations of this hormone can be found at the junction between the portal vein and the liver parenchyma [4-6], which is also the liver entry site of the oncosphere [2].

Due to its important role in regulating a variety of metabolic and developmental processes, insulin signalling has been well studied in mammals and invertebrate model systems, such as *Caenorhabditis elegans* and *Drosophila melanogaster*[7-9]. Insulin signalling is initiated by binding of insulin-like hormones to surface receptor tyrosine kinases of the insulin/insulin-like growth factor (IGF) family that are usually produced as long pro-peptides which are later processed into an extracellular α-subunit and a membrane-spanning β-subunit, connected by a disulphide bridge [7-9]. Upon ligand binding to surface associated α2β2 receptor tetramers, auto-phosphorylation of several tyrosine residues within the β-subunit is induced, one of which forms part of a well conserved NPXY-motif that is located in the juxta-membrane region [7-9]. Downstream signalling is then induced by binding of intracellular adapter proteins (for example, insulin receptor substrate; IRS) to the phosphorylated NPXY motif. The two major downstream signalling pathways in vertebrates and invertebrates are the ERK1/2 mitogen-activated protein kinase (MAPK) cascade and the phosphoinositide-3-kinase (PI3K)/protein kinase B (PKB; also known as Akt) pathway [7-9].

Insulin signalling mechanisms have already been studied to a certain extent in parasitic and free-living flatworms. Tyrosine kinases of the insulin receptor family have been fully characterized in the cestode *E. multilocularis*[10] and the trematodes *Schistosoma mansoni*[11,12] and *S. japonicum*[13]. Using the yeast two-hybrid system it was further shown that the ligand binding domains (LBD) of the flatworm insulin receptor tyrosine kinases are principally able to bind human insulin [10,11,13], although it is not yet clear whether they are also activated by insulin when expressed at the parasite surface. Reduced glucose uptake in *in vitro* cultivated schistosomes upon treatment with insulin receptor inhibitors indicated that, at least in trematodes, insulin signalling might regulate glucose homeostasis [12]. Several investigations on the direct influence of host insulin on flatworm parasite glucose uptake and/or development showed slight effects and were carried out using un-physiologically high concentrations of the host hormone [12,14,15]. Finally, a very recent study in the free-living model system *Schmidtea mediterranea* demonstrated a role of insulin signalling in the regulation of flatworm stem cell activity and proliferation [16].

During recent years, we have developed several cultivation systems by which the developmental transitions of *E. multilocularis* larvae within the intermediate host can be mimicked *in vitro*[2,17-19]. These include systems for investigating proliferation and differentiation of metacestode vesicles under host cell free conditions [18] as well as a parasite stem cell cultivation system that closely mimics the metamorphic transition of the oncosphere towards the metacestode [19,20]. Using these systems we addressed, in the present study, questions on the influence of physiological concentrations of human insulin on parasite development, glucose uptake and the activation of *Echinococcus* insulin signalling pathways. We demonstrate that *E. multilocularis* larval development is significantly stimulated in the presence of physiological concentrations of human insulin, and that the parasite’s insulin signalling pathways are activated upon exogenous addition of insulin. We also show that the *E. multilocularis* insulin signalling pathways are affected by an insulin receptor inhibitor originally designed against the human insulin receptor and that this treatment results in impaired larval development and parasite killing.

## Results

### Host insulin stimulates *E. multilocularis* larval development *in vitro*

**Figure 1:**
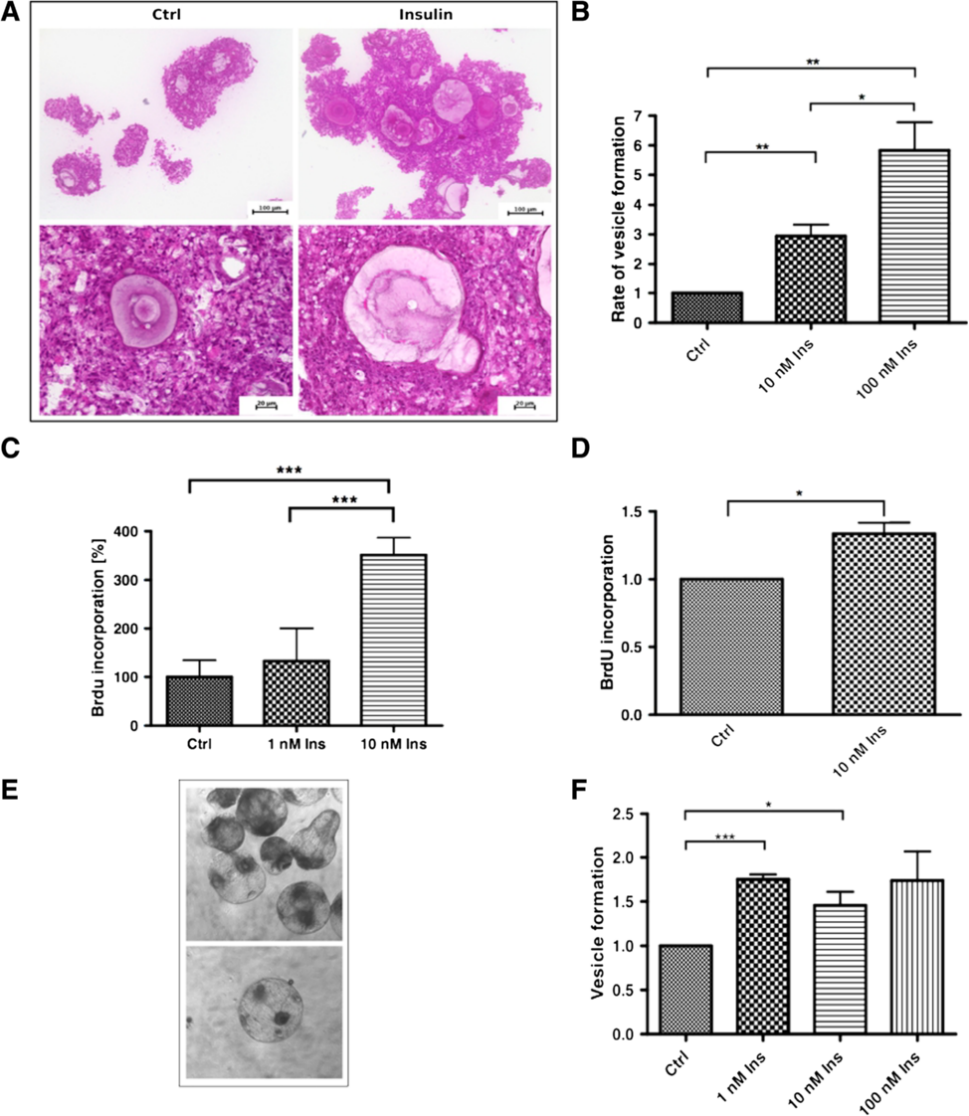
**Effects of insulin on *E. multilocularis *larval development. A)** Morphology of primary cell aggregates. Primary cells were isolated from axenic metacestode vesicles and cultivated in 2% FCS/(D)MEM supplemented with or without 10 nM human insulin for one week. The aggregates were fixed and embedded in Technovit 8100. Sections (4 μm) were stained with haematoxylin/eosin. **B)** Formation of metacestode vesicles. Primary cells were cultivated in conditioned medium supplemented with human insulin for three weeks and mature metacestode vesicles were counted. Control was set to 1 and results were normalised against the control. (*) *P* values below 0.05, (**) very significant for *P* between 0.001 and 0.01, (***) extremely significant for *P* <0.001. **C)** BrdU uptake by parasite cell cultures. Primary cells were isolated and incubated for 24 hours with insulin. BrdU was added for four hours and BrdU uptake was measured with the colorimetric BrdU ELISA kit (Roche, Mannheim, Germany). Asterisks mark significant values. **D)** BrdU uptake by mature metacestode vesicles. Metacestode vesicles were incubated for two days in the presence or absence of insulin and BrdU. BrdU uptake was measured after chromosomal DNA isolation with the colorimetric BrdU ELISA kit (Roche). **E)** Re-differentiation and microcyst formation of *E. multilocularis* protoscoleces. Examples of microcysts forming in *in vitro* protoscolex cultures. **F)** Microcyst formation of *in vitro* cultivated protoscoleces incubated with insulin for three weeks. Control was set to 1 and results were normalised against the control. (*) *P* values below 0.05, (**) very significant for *P* between 0.001 and 0.01, (***) extremely significant for *P* <0.001. BrdU, bromodeoxyuridine.

To study the influence of human insulin on parasite development, three different *in vitro* cultivation systems were used. First, we studied the effect of insulin on isolated *E. multilocularis* primary cells that contain high numbers of totipotent stem cells, which lead to the formation of metacestode vesicles in a manner that closely resembles the oncosphere-metacestode transition during the early phase of *in vivo* infections [19,20]. As depicted in Figure 1A, parasite cell aggregates that result from stem cell proliferation [19] were always larger in insulin treated samples and, when compared to the control, also contained larger internal cavities which later gave rise to mature vesicles. The formation of mature vesicles was also significantly stimulated and approximately three-fold and six-fold more vesicles were detected after one week incubation in the presence of 10 nM and 100 nM insulin, respectively (Figure 1B). Although insulin-treated samples consistently yielded higher numbers of mature metacestode vesicles, the eventually obtained vesicle size in these samples was not significantly enlarged when compared to the controls (data not shown). As displayed in Figure 1C, insulin-treatment also significantly stimulated the uptake of bromodeoxyuridine (BrdU) in parasite primary cell cultures, indicating that the host hormone has a direct effect on the proliferation rate of parasite stem cells, which are the only cells capable of proliferation in flatworms [2].

We next tested the effects of host insulin on the development of mature metacestode vesicles. Although insulin-treatment showed a clear trend to yield larger vesicles after about two weeks of incubation (data not shown), measurement of parasite development on the basis of vesicle volume increase is difficult in this system. We, therefore, mainly tested stem cell proliferation and, as shown in Figure 1D, insulin treatment significantly stimulated BrdU uptake in metacestode vesicles, although not as prominently as in the case of primary stem cell cultures.

Protoscoleces of the closely related dog-tapeworm *E. granulosus* show the unique capacity of being able to mature into strobilar adult stages, when ingested by a definitive canid host, but also of ‘re-differentiating’ into fully developed cysts when released into the intermediate host body cavity upon cyst rupture [3]. This capacity appears to be also shared by protoscoleces of *E. multilocularis*[21]. To investigate the effects of host insulin on the *Echinococcus* re-differentiation processes, we employed a cultivation system in which *E. multilocularis* protoscoleces were kept in the presence of hepatocyte-conditioned medium that usually induces vesicle formation from parasite stem cells [19]. As shown in Figure 1E, *E. multilocularis* protoscoleces did indeed re-differentiate into fully mature metacestode vesicles under these conditions, although the number of protoscoleces that underwent re-differentiation was usually very low (about 2.2% of all protoscoleces in culture). In the presence of 1 nM or 10 nM insulin, however, the number of fully re-differentiated protoscoleces was significantly increased by around 50% (Figure 1F).

Taken together, these results indicated that *E. multilocularis* stem cell cultures and larvae are responsive to physiologically relevant concentrations of host insulin and that this treatment stimulates the formation of metacestode vesicles either from stem cell cultures or from protoscoleces.

### Characterization of *E. multilocularis* insulin-receptors

**Figure 2:**
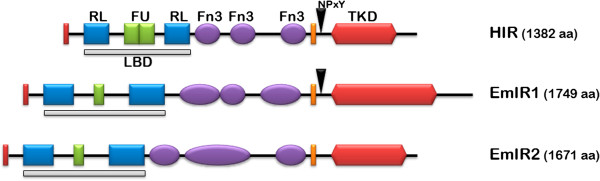
**Domain structure of *E. multilocularis *insulin receptors.** Schematic representation of the domain structures of the human insulin receptor (HIR), EmIR1 and EmIR2 according to SMART analyses (Letunic *et al*., 2012). Displayed are the location and size of the following predicted domains: TKD, tyrosine kinase domain; LBD, ligand binding domain; RL, receptor-L-domain; FU, furin-rich repeat; Fn3, fibronectin 3 domain. The presence of NPXY-motifs for binding of IRS is indicated by an arrowhead. Red bars at the N-terminus represent signal peptides, orange bars represent transmembrane domains. IRS, insulin receptor substrate; SMART, Simple Modular Architecture Research Tool.

We had previously characterized an insulin-like receptor tyrosine kinase of *E. multilocularis*, originally designated EmIR, which at least in the yeast two-hybrid system interacted with human pro-insulin [10]. When analysing the recently released whole genome sequence of *E. multilocularis*[22] the respective gene, *emir*, was identified on scaffold 7780 (gene ID: EmuJ_000962900) and comprised 25 exons, separated by 24 introns, as previously determined [10]. In extensive Basic Local Alignment Search Tool (BLAST) analyses using the amino acid sequences of the human insulin receptor (HIR), EmIR, and previously described insulin receptors from schistosomes [11-13], we found a second gene on the *E. multilocularis* genome that obviously encoded another receptor tyrosine kinase of the insulin receptor family. The respective gene was designated *emir2* (scaffold 7780; gene ID: EmuJ_000981300; 24 exons) and the previously identified gene, *emir*, was re-named to *emir1*. The entire *emir2* cDNA was cloned and sequenced and found to encode a protein, EmIR2, of 1,671 amino acids (181 kDa). In Simple Modular Architecture Research Tool (SMART) homology analyses, EmIR2 displayed a domain structure typical of insulin receptor tyrosine kinases, with a predicted signal peptide, a LBD composed of two receptor L domains separated by a furin domain, three fibronectin 3 domains, a transmembrane domain and an intracellular tyrosine kinase domain (TKD) (Figure 2). Particularly within the TKD and the LBD, EmIR2 showed significant amino acid sequence homologies to EmIR1 and insulin receptors of mammalian and schistosome origin [see Additional file 2]. Interestingly, and in contrast to EmIR1, EmIR2 did not contain a NPXY motif in the juxtamembrane region which, in HIR, is important for downstream signalling through IRS [7-9] (Figure 2).

**Figure 3:**
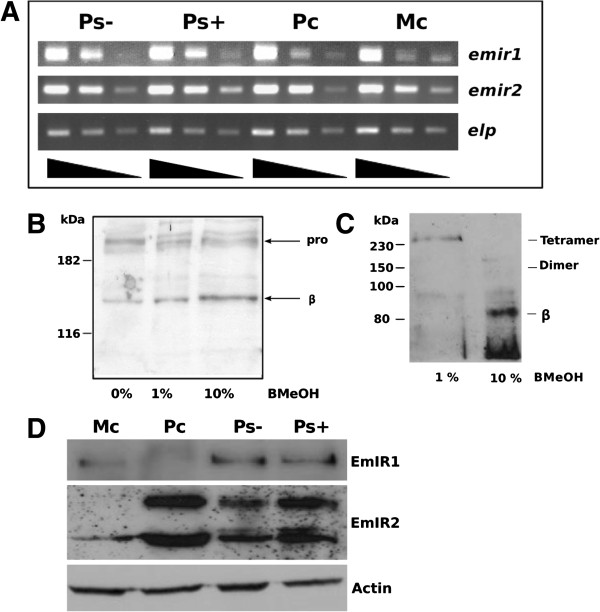
**Expression of EmIR1 and EmIR2 in *E. multilocularis *larval stages. A)** Semi-quantitative RT-PCR of *emir1* and *emir2* expression. Serial 1/10 dilutions of cDNA from metacestode vesicles (MC), primary cells (PC) as well as dormant (PS-) and low pH/pepsin-activated protoscoleces (PS+) were subjected to gene-specific PCR using intron-flanking primers. PCR products were separated on a 1% agarose gel and stained with ethidium bromide. The constitutively expressed gene *elp* was used as control. **B)** Western blot and immunoprecipitation employing the EmIR1 anti-serum. EmIR1 was immunoprecipitated from metacestode vesicles and treated with β-mercaptoethanol (beta-MeOH) at concentrations of 0%, 1% and 10%. Probes were then separated on a 12.5% polyacrylamide gel and developed using the anti-EmIR1 antiserum. ‘pro’ and ‘beta’ indicate the pro-form and the β-subunit of EmIR, respectively. **C)** Immunoprecipitation and Western blot using the anti-EmIR2 serum. EmIR2 was immunoprecipitated from protoscolex preparations, samples were then supplemented with 1% or 10% of β-mercaptoethanol (β-ME) and separated on a 10% SDS gel. Western blot was carried out using the anti-EmIR2 antiserum. **D)** Immunodetection of EmIR1 and EmIR2 in different larval stages using immune sera. Parasite larvae were lysed, protein preparations were then separated by SDS-PAGE, blotted onto a membrane and detected by the antisera. The purified anti-EmIR2 immune serum recognized the EmIR2 β-subunit at 87 kDa and a second band at 60 kDa. The EmIR1 β-subunit was detected at 150 kDa using the anti-EmIR1 immune serum. Actin was used as a loading control. Mc, metacestode vesicles; Pc, primary cells; Ps-, dormant protoscoleces; Ps+, activated protoscoleces.

We considered both EmIR1 and EmIR2 likely candidates for mediating the effects of host insulin on the parasite larval stages and, thus, analysed the role of these kinases in *Echinococcus* insulin signalling in more detail. First, we carried out semi-quantitative RT-PCR experiments to analyse the expression patterns of the *Echinococcus* insulin receptor genes in different larval stages. As shown in Figure 3A, *emir1* and *emir2* expression was detected in all larval stages that were responsive to host insulin. We also analysed the expression levels of both genes in available transcriptome data sets that were generated for gene annotation of the *E. multilocularis* genome project [22]. According to these data, *emir1* is about two- to three-fold higher expressed in *Echinococcus* larvae than *emir2,* and both genes show the lowest expression levels in adult worms [see Additional file 3]. For biochemical and histochemical investigations, we next produced specific antisera directed against the intracellular portions of EmIR1 and EmIR2 [see Additional file 4]. As shown in Figure 3B, the anti-EmIR1 antiserum detected a band of approximately 150 kDa, the intensity of which increased upon treatment of parasite lysate with β-mercaptoethanol, as well as several larger bands around 195 kDa. This pattern indicated that the 150 kDa band represents the EmIR1 β-subunit, whereas the 195 kDa band(s) are most likely αβ-subunit dimers that are still connected by disulphide bridges. The actual molecular mass of the EmIR1 β-subunit is higher than the calculated mass of the polypeptide (approximately 100 kDa), which is most probably due to post-translational modification, such as glycosylation, as has already been shown for insulin receptor β-subunits of other organisms, including the human insulin receptor [23]. In the case of EmIR2, an intense band of 87 kDa was observed when immunoprecipitates were treated with 10% β-mercaptoethanol, indicating that this is the EmIR2 β-subunit, whereas in the presence of 1% β-mercaptoethanol one large band was visible that, due to its size of >230 kDa, could represent an α2β2 tetramer. When total parasite lysate was probed with the anti-EmIR2 antiserum, a smaller band of approximately 60 kDa was detected alongside the 87 kDa band, which could be due to alternative processing of the EmIR2 β-subunit (Figure 3D).

Interestingly, when we analysed the *E. multilocularis* larval stages for the presence of EmIR1 in Western blot experiments, clear signals were obtained for protoscoleces and metacestode vesicles but no signal was obtained for primary cell cultures (Figure 3D). In the case of EmIR2, on the other hand, signals were obtained for protoscoleces and primary cells, but only a very faint signal was seen in metacestode preparations (Figure 3D). Since RT-PCR and transcriptome data revealed the presence of *emir1* transcripts in primary cell cultures and *emir2* transcripts in metacestode vesicles (see above), these results were unexpected and indicated that the expression of EmIR1 and EmIR2 in primary cell cultures and metacestode vesicles, respectively, might be subject to translational repression.

**Figure 4:**
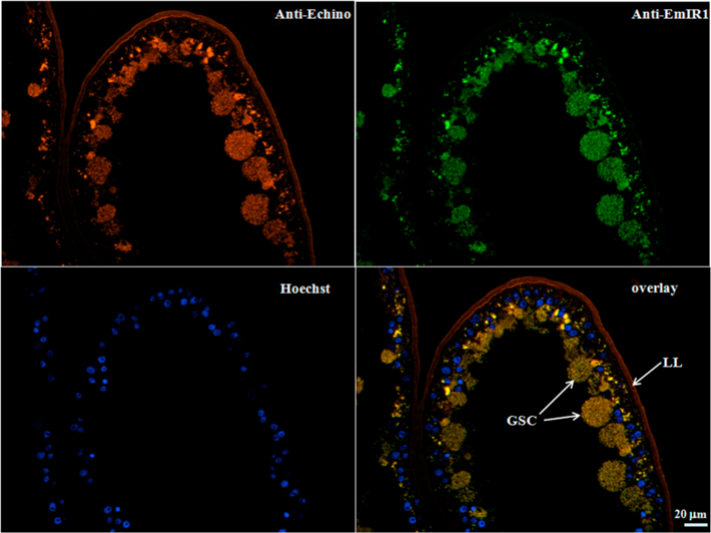
**EmIR1 immunohistochemistry on metacestode vesicles.** Cryosections of *in vitro* cultivated metacestode vesicles were probed with the anti-EmIR1 antiserum (anti-EmIR1) and detected with a FITC-coupled anti-rabbit-antibody. Nuclei were visualized by Hoechst-staining (Hoechst). Parasite surface structures were visualized using a general anti-*Echinococcus* metacestode antibody (Anti-Echi; Ingold *et al*., 2001 [50]). LL, laminated layer; GSC, glycogen storing cells. FITC, fluorescein isothiocyanate.

**Figure 5:**
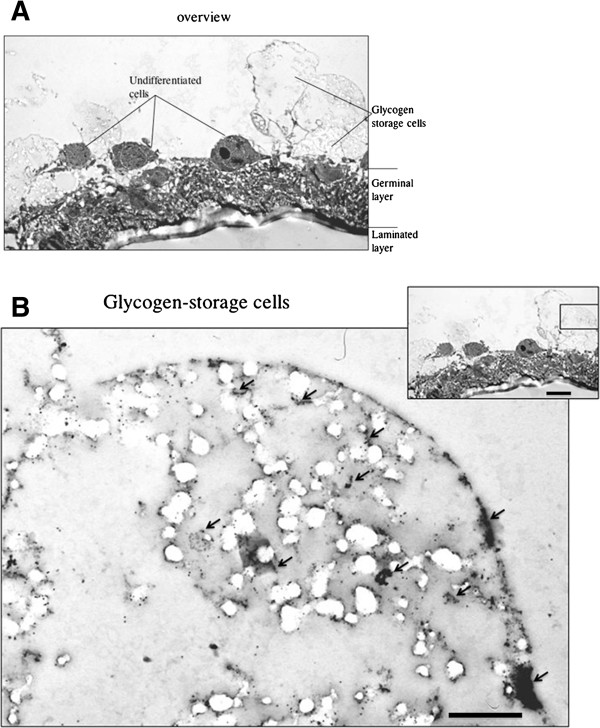
**EmIR1 electron microscopic analyses.** The anti-EmIR1 antiserum and gold-coupled anti-rabbit antibodies were used to detect EmIR1 in the metacestode germinal layer. **A)** Overview showing the location of the laminated layer, the germinal layer, undifferentiated (stem) cells and glycogen storage cells. **B)** Glycogen storage cell showing massive anti-EmIR1 staining (arrows). Scale bar in larger image represents 0.6 μm, scale bar in insert represents 3.6 μm.

**Figure 6:**
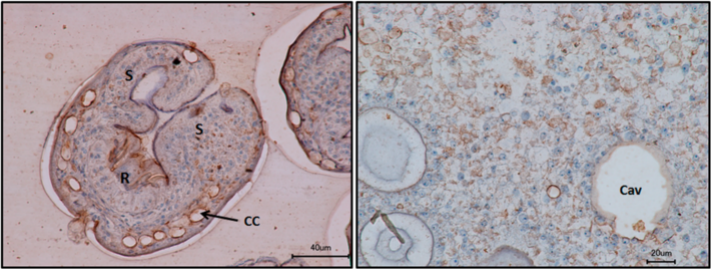
**Anti-EmIR2 immunohistochemistry.** Sections of *E. multilocularis* protoscolex preparations (left panel) and primary cell culture aggregates (right panel) have been probed with the anti-EmIR2 antiserum. Brown color indicates positive signals. Cav, central cavity; S, suckers; R, rostellum; CC, calcareous corpuscles.

Using the anti-EmIR1 antiserum, we next investigated the localization of EmIR1 in *Echinococcus* larval stages by immunohistochemistry, immunofluorescence and electron microscopy. As expected from the Western blot experiments mentioned above, no EmIR1 staining was obtained for primary cell cultures (data not shown). Most strikingly, however, we observed particularly strong staining for a population of large, round cells present at the proximal layer of the metacestode (Figure 4). These cells clearly represented the parasite’s glycogen storing cells (GSC), in which glycogen is not preserved when fixed without tannic acid. These results could be verified by transmission electron microscopy using immune-gold labelled anti-EmIR1 antiserum. Again, strong signals were obtained for GSC (Figure 5), but weaker signals associated with other cell types, such as undifferentiated parasite stem cells, were also observed (data not shown). For EmIR2, staining of the parenchyma close to the surface of the protoscolex was obtained as well as a diffuse staining pattern throughout primary cell aggregates (Figure 6). For metacestode vesicles, on the other hand, no EmIR2 signal was obtained (data not shown).

**Figure 7:**
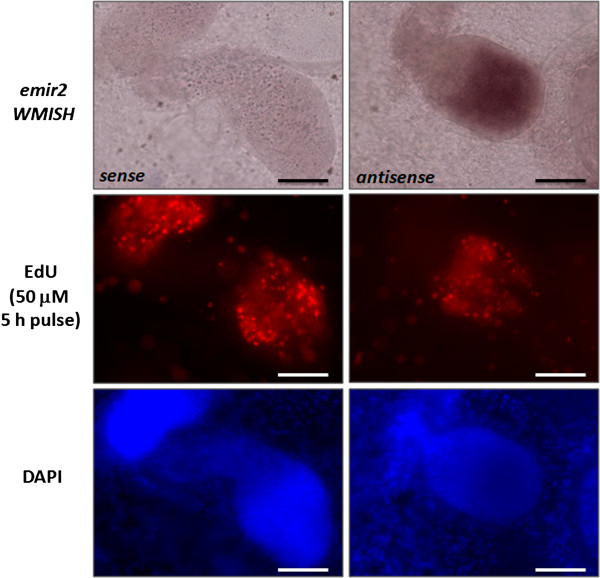
**Whole mount *in situ *hybridisation (WMISH) detection of *emir2*.** Germinal layer with developing protoscoleces stained for *emir2* WMISH (upper panel), EdU incorporation (mid panel) and DAPI (lower panel). Sense (left) and antisense (right) probes have been used as indicated. Bar represents 50 μm. DAPI, 4',6-diamidino-2-phenylindole; EdU, 5-ethylnyl-2’-deoxyuridine.

Using an established protocol applicable to metacestode vesicles, we also investigated *emir2* expression by *in situ* hybridisation. In these experiments, no signal was obtained for the germinal layer of vesicles that had not yet started to develop protoscoleces (data not shown). However, in fertile vesicles an intense *emir2* signal was associated with the proliferation zone of developing protoscoleces, in which parasite stem cells undergo cell division, as indicated by the incorporation of the thymidine analogue 5-ethylnyl-2’-deoxyuridine (EdU) (Figure 7). These data indicated that *emir2* might be specifically expressed in parasite stem cells or, at least, in parasite tissues that are actively proliferating.

Taken together these results demonstrated that EmIR1 is present in metacestode vesicles where it is primarily associated with GSC. Since host insulin is present not only outside of metacestode vesicles but highly likely also within hydatid fluid, which contains a large number of host serum proteins [24], the localisation of EmIR1 also suggests that it has direct contact with host insulin. EmIR2, on the other hand, was not present in metacestode vesicles (or at concentrations below the detection limit of the staining method), but dispersed throughout primary cell aggregates. Furthermore, *emir2* expression was prominent in developing protoscoleces which suggests an association with parasite stem cells.

### Host insulin stimulates glucose uptake by metacestode vesicles

**Figure 8:**
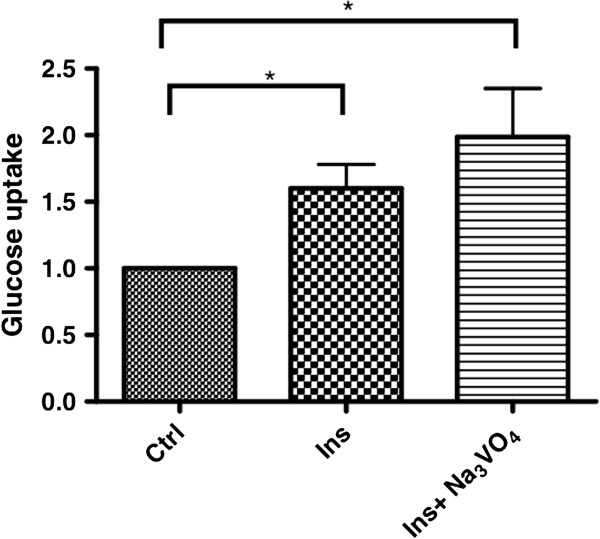
**Effects of insulin on metacestode glucose uptake.** Uptake of C^14^-D-glucose by metacestode vesicles in the presence of 10 μM insulin alone,and insulin together with Na_3_VO_4_. Control was set to 1 and results were normalised against the control. (*) *P* values below 0.05, (**) very significant for *P* between 0.001 and 0.01, (***) extremely significant for *P* <0.001.

The prominent localisation of EmIR1 in GSC indicated that this receptor could be involved in *Echinococcus* glucose uptake/storage mechanisms. To investigate this aspect, metacestode vesicles were cultivated in the presence of radioactively labelled glucose and were either stimulated with host insulin or not. As shown in Figure 8, the addition of 10 nM insulin to metacestode vesicles significantly stimulated glucose uptake after one hour of incubation, which was even more pronounced after addition of the phosphatase inhibitor Na_3_VO_4_, indicating that phosphorylation events are involved in regulating *Echinococcus* glucose uptake or transport. Hence, similar to the situation in intermediate host hepatocytes, insulin also regulates glucose uptake by parasite cells.

### Host insulin affects parasite signalling pathways

**Figure 9:**
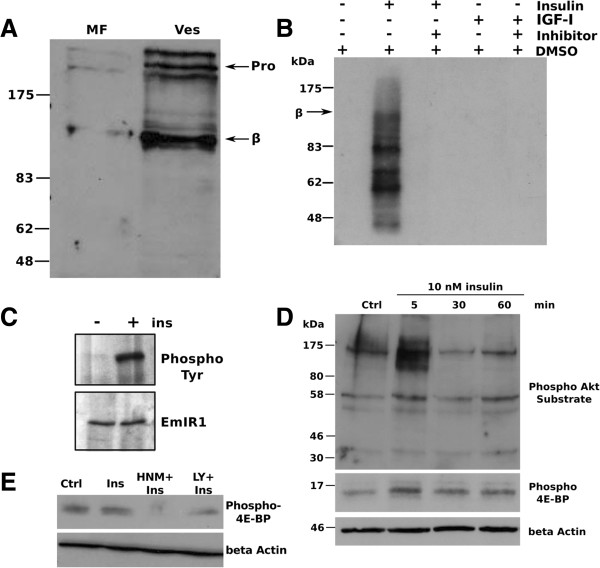
**Effects of insulin on the phosphorylation of metacestode vesicle proteins. A)** Detection of EmIR1 in vesicle membrane fractions (MF). Vesicles from *in vitro* culture were homogenized and the MF was isolated. Western blot detection was carried out on MF and whole vesicle preparations (Ves) using the anti-EmIR1 antiserum. ‘Pro’ and ‘β’ mark the receptor pro-form and β-subunits. **B)** Phosphorylation of EmIR1 in response to insulin. Metacestode MF was stimulated for 10 minutes with either 100 nM insulin or IGF-I, followed by 30 minutes incubation with 100 μM HNMPA(AM)_3_ or control DMSO. Phosphorylation of membrane proteins was carried out for 40 minutes in the presence of [^32^P] γ-ATP. Proteins of the MF were precipitated using the anti-EmIR1 antiserum. Proteins were then separated by 8% SDS-PAGE and phosphorylation was detected by autoradiography. Bands are visible at the size of the EmIR1 β-subunit and below. **C)** Phosphorylation of EmIR1 after insulin stimulation of metacestode vesicles. Vesicles were stimulated (+) or not (−) with 100 nM insulin for 10 minutes. Following solubilisation of membrane proteins, the EmIR1 β-subunit was precipitated using the anti-EmIR1 antiserum and separated by SDS-PAGE. Western blot detection was carried out using the anti-EmIR1 antiserum (lower panel) or an anti-phospho-tyrosine antibody (upper panel). **D)** Phosphorylation of *Echinococcus* PI3K/Akt pathway components in response to insulin. Vesicles were stimulated with 10 nM insulin for the times indicated above. Vesicle lysates were subsequently separated by SDS-PAGE and probed using antibodies against the phosphorylated Akt substrate motif or phospho 4E-BP as indicated. β-Actin was used as loading control. **E)** Inhibition of 4E-BP phosphorylation through HNMPA(AM)_3_. Vesicles were incubated for two hours with 100 μM HNMPA(AM)_3_ (HNM+) or the PI3K inhibitor LY294002 (LY+) before stimulation with 10 nM insulin. Crude lysates were probed with the anti-phospho 4E-BP antibody. β-Actin was used as loading control. DMSO, dimethyl sulphoxide; HNMPA, 2-hydroxynaphthalen-1-yl-methylphosphonic acid.

Next, we investigated parasite signalling pathways that could be involved in insulin sensing and signalling. First, we concentrated on EmIR1, which showed prominent expression in GSC of metacestode vesicles. The membrane fraction of metacestode vesicles was isolated and the presence of EmIR1 in this fraction was verified by Western blotting (Figure 9A). The proteins in the membrane fraction were then stimulated with human insulin for 30 minutes in the presence of [^32^P]- γATP. As a control, human IGF was tested which, unlike insulin, has previously been shown not to interact with the EmIR1 LBD in yeast two-hybrid experiments [11]. After stimulation, EmIR1 was immunoprecipitated from the membrane fraction and phosphorylation was analysed by gel electrophoresis and autoradiography (Figure 9B). In order to ensure maximal activation of the receptor in this complex immunoprecipitation experiment, saturating hormone concentrations of 100 nM were used, as usual for comparable experiments on insulin receptors [25-30]. As depicted in Figure 9B, EmIR1 protein phosphorylation was detected after stimulation with insulin, but not with IGF. Protein bands smaller than the expected size of the EmIR1 β-subunit (Figure 9B) were most probably due to degradation of membrane proteins in this experimental setting.

We also employed a specific compound, HNMPA(AM)_3_, that was originally designed to inhibit the human insulin receptor [13] and was subsequently found to also inhibit schistosome insulin receptors [13,31]. To confirm that HNMPA(AM)_3_ is principally also able to inhibit EmIR1, *in silico* modelling of the EmIR1 TKD was carried out and revealed that HNMPA(AM)_3_ has comparable affinities for the EmIR1 TKD and the HIR TKD [see Additional file 5]. Interestingly, when stimulated by insulin in the presence of 100 μM HNMPA(AM)_3_, no phosphorylation of EmIR1 immunoprecipitates was observed (Figure 9B), indicating that this compound prevents EmIR1 activation.

We also investigated specific tyrosine phosphorylation of EmIR1 after insulin treatment of metacestode vesicles. To this end, intact *in vitro* cultivated metacestode vesicles were stimulated with 100 nM insulin (to ensure maximum activation) for 10 minutes, followed by immunoprecipitation of EmIR1, using the anti-EmIR1 antiserum. Subsequently, detection of phosphorylated tyrosine using an anti-phospho-tyrosine antibody was carried out. As shown in Figure 9C, insulin treatment indeed induced tyrosine phosphorylation of the EmIR1 β-subunit in these vesicles.

In other organisms, signalling pathways that act downstream of insulin receptors usually involve phosphorylation of several intracellular signalling factors. Among the signalling pathways that are stimulated by insulin in many organisms is the ERK-like MAPK cascade [7-9], a complete module of which we had previously characterized in *E. multilocularis*[32,33]. When we analysed the activation of the *Echinococcus* MAPK cascade in response to exogenously added insulin by measuring phosphorylation of the ERK-like MAPK EmMPK1 [32], however, only a very weak induction was observed (data not shown). We, therefore, concentrated in additional experiments on the PI3K/Akt-pathway, another major downstream target of insulin signalling in vertebrates and invertebrates [7-9]. So far, this pathway had not been addressed in *Echinococcus* or other parasitic helminths. We, therefore, first screened the available *E. multilocularis* genome sequence [22] and could indeed identify genes encoding several key components of this pathway, such as a catalytic subunit of PI3K, an ortholog to mTOR (mammalian target of rapamycin), a glycogen synthase kinase-ortholog, and orthologs to protein kinase B (also called Akt kinase) or the eukaryotic translation initiation factor 4E-binding protein (4E-BP) [see Additional file 6]. We were particularly interested in the genes encoding the *E. multilocularis* orthologs of Akt [see Additional file 7] and 4E-BP [see Additional file 8] and fully cloned and sequenced the respective cDNAs. Next, we used antibodies that either detect the phosphorylated form of the evolutionarily conserved Akt kinase target motif RxRxxS/T or the phosphorylated form of 4E-BP in a region that is highly conserved among orthologs of different species [see Additional file 8] to study the effects of insulin on the PI3K/Akt pathway. As depicted in Figure 9D, some basic level of phosphorylation was detected both for Akt substrates and 4E-BP, which is most probably due to the fact that serum-containing media inevitably contain residual concentrations of insulin, which cannot be completely removed. However, particularly after a five minute treatment of metacestode vesicles with 10 nM exogenous insulin, a marked phosphorylation of several additional proteins could be observed using the anti-phospho-Akt-substrate antibody. Furthermore, using the anti-phospho-4E-BP antibody, a clearly enhanced phosphorylation of a single protein with a molecular mass within the range of 4E-BPs was detected. We then also investigated whether 4E-BP phosphorylation in response to insulin can be inhibited by HNMPA(AM)3 (pre-incubation with 100 μM inhibitor) and found that this was indeed the case (Figure 9E).

Taken together, these results indicated that exogenously added insulin directly stimulated EmIR1 in intact metacestode vesicles and that insulin treatment also led to an activation of the PI3K/Akt-pathway in *Echinococcus*.

### An insulin receptor inhibitor blocks parasite development *in vitro*

**Figure 10:**
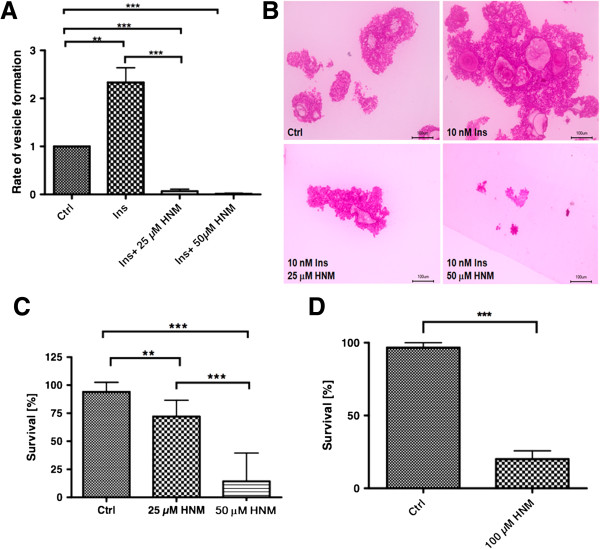
**Effects of HNMPA(AM)**_**3 **_**on parasite larvae. A)** Primary cells were isolated from axenic metacestode vesicles and cultivated in 2% FCS/(D)MEM supplemented with 10 nM human insulin, with or without HNMPA(AM)_3_. After three weeks of incubation, mature vesicles were counted. Insulin was added to the cultures in order to obtain vesicle formation within three weeks. **B)** Formation of primary cell aggregates in the presence of HNMPA(AM)_3._ Primary cells were incubated as in (A) for seven days before aggregates were fixed, embedded in Technovit 8100 and 4 μm sections stained with haematoxylin/eosin. Note the profound effect of HNMPA(AM)_3_ on parasite aggregates already after seven days. Ctrl, DMSO control. **C)** Protoscoleces were treated with HNMPA(AM)_3_ for two weeks under axenic conditions. Protoscolex viability was analysed by counter-staining with methylene blue. **D)** Metacestode vesicles were treated for one week with 100 μM HNMPA(AM)_3_ under axenic conditions. Survival was assessed by counting physically damaged vesicles. Vesicles were incubated in the presence of conditioned medium for optimal maintenance and survival conditions. (*) *P* values below 0.05, (**) very significant for *P* between 0.001 and 0.01, (***) extremely significant for *P* <0.001. DMSO, dimethyl sulphoxide; HNMPA, 2-hydroxynaphthalen-1-yl-methylphosphonic acid.

Since the insulin receptor inhibitor HNMPA(AM)_3_ prevented the phosphorylation of EmIR1 in metacestode membrane fractions (see above), we further investigated the effects of this small molecule compound on parasite development. First, we investigated larval development from stem cell cultures to metacestode vesicles and observed that vesicle formation was almost completely abolished at concentrations of 25 and 50 μM of HNMPA(AM)_3_ (Figure 10A,B). These concentrations also significantly decreased protoscolex viability (Figure 10C), but were ineffective in killing metacestode vesicles, at least after seven days of incubation (data not shown). In the presence of 100 μM HNMPA(AM)_3_, on the other hand, only 20% of metacestode vesicles survived after seven days (Figure 10D). Taken together, these data indicated that intact insulin receptor signalling is important for parasite survival and development.

### Characterization of insulin-like peptides in *E. multilocularis*

**Figure 11:**
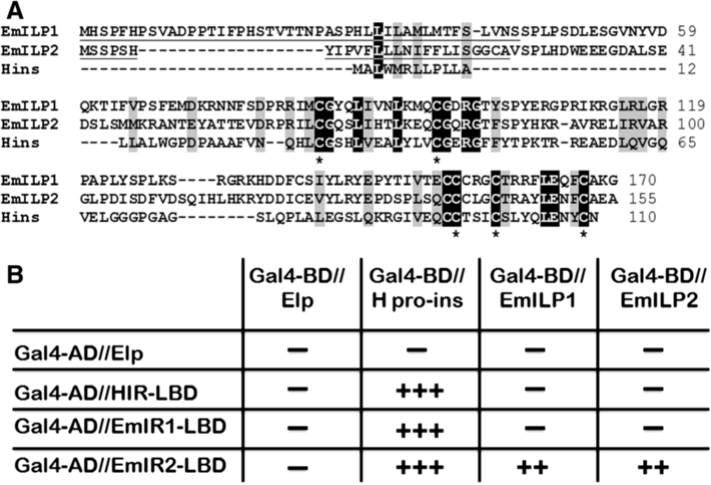
***E. multilocularis *insulin-like peptides and yeast two-hybrid experiments. A)** Amino acid sequence comparison between the *E. multilocularis* insulin-like peptides EmILP1 and EmILP2 and human insulin (Hins). Highly conserved residues are printed in white on black background, residues with similar biochemical features are printed in black on grey background. Underlined sequences indicate predicted signal peptides. Asterisks indicate cysteine residues important for disulphide-bridge formation. **B)** Yeast two-hybrid experiments. Translational fusions were generated for the Gal4 activation domain (Gal4-AD) and the LBDs of the human insulin receptor (HIR-LBD) as well as the *E. multilocularis* receptors EmIR1 (EmIR1-LBD) and EmIR2 (EmIR2-LBD). The Gal4 DNA binding domain (Gal4-BD) was fused to human pro-insulin (H pro-ins) as well as to EmILP1 and EmILP2. Yeast strains were double transformed with the plasmid constructs as indicated and growth under different stringency conditions [10] was assessed. ‘-‘ indicates no growth, ‘++’ growth under medium stringency conditions, ‘+++’ growth under high stringency conditions. LBDs, ligand binding domains.

As yet, the presence of genes that encode insulin-like peptides has been described for the free-living flatworm *S. mediterranea*[16] but not in any parasitic flatworm. We, therefore, screened the *E. multilocularis* genome [22] by BLAST analyses for the presence of such genes. Indeed, we found two genes located immediately adjacent to each other (separated by 17 kb) on contig 60709 of the current assembly, which code for peptides with moderate overall homology to human insulin, but which display classical signatures of insulin-like peptides (ILPs) (Figure 11A). Both genes, named *emilp1* (EmuJ_000045300) and *emilp2* (EmuJ_000045400), code for peptides that, according to SMART analyses, contain an IIGF domain (insulin/insulin-like growth factor/relaxin) and an export-directing signal peptide (Figure 11A), indicating that they are secreted. By RT-PCR analyses it was very difficult to amplify *emilp1* and *emilp2* transcripts from RNA preparations of primary cells, metacestode vesicles and protoscoleces (data not shown), indicating that both genes are very slightly expressed in parasite larval stages. Accordingly, in available transcriptome data sets for *E. multilocularis*[22], *emilp1* and *emilp2* show highest expression levels in the adult stage [see Additional file 3], whereas only moderate expression was found for *emilp2* in parasite larvae and no expression of *emilp1* in primary cells and metacestode vesicles [see Additional file 3].

The interaction between ILPs and cognate receptors has previously been investigated using the yeast two-hybrid system [10,11,13] and we employed this approach to also study interactions between human insulin, the parasite ILPs and possible cognate receptors. As shown in Figure 11B, human pro-insulin interacted strongly with the LBD of HIR and both parasite insulin receptors. EmILP1 and EmILP2, on the other hand, only showed detectable interaction with EmIR2, whereas none of the parasite ILPs interacted with EmIR1 or HIR.

Taken together, these analyses demonstrate that the *E. multilocularis* genome encodes ILPs, but the respective genes appear to be mainly involved in developmental processes of the adult stage which resides in the definitive host’s gut and, thus, has no access to host-derived insulin. As previously shown [10], human insulin could interact with EmIR1 and we now demonstrated that it also interacts with the LBD of EmIR2. Finally, of all ILPs tested, only human insulin appeared capable of acting as a ligand for EmIR1.

## Discussion

Since the initial characterization of a member of the insulin receptor family in *E. multilocularis*, EmIR1 [10], few studies have been conducted to investigate the effects of mammalian insulin on flatworm parasite insulin signalling pathways and development. In each of the related parasites *Schistosoma mansoni* and *S. japonicum*, two EmIR1-like tyrosine kinases of the insulin receptor family were identified and, as originally shown for EmIR1, the possibility of an interaction of these receptors with host insulin was verified using the yeast two-hybrid system [11,13]. These studies did, however, not address whether host-derived insulin would stimulate (or generally influence) parasite development and/or establishment within the host. Although Ahier *et al*. [12] later investigated effects of host insulin on glucose uptake of *S. mansoni in vitro*, significant stimulation was only achieved using hormone concentrations of 1 μM, which can be considered non-physiologically high since plasma levels of insulin in humans and animals usually range between 1 to 2 nM [34,35]. Likewise, in studies on cestode systems conducted by Canclini and Esteves [15] (*Mesocestoides corti*) and Escobedo *et al*. [14] (*Taenia crassiceps*), effects on glucose metabolism or parasite development (*T. crassiceps* budding) were only observed at insulin concentrations several magnitudes higher than physiological concentrations. Hence, although several investigations had already addressed the possibility of insulin-based hormonal cross communication between flatworm parasites and mammalian hosts, it is still unclear to date whether host insulin at physiological concentrations indeed influences parasite development and metabolism or whether such effects are mediated by evolutionarily conserved insulin signalling systems of these parasites.

In the present study, we concentrated on a cestode, *E. multilocularis*, the larval stage of which displays a strong organ-tropism towards the liver where the highest insulin concentrations (up to 15 nM) within mammals can be measured [4-6]. Several independent lines of evidence clearly indicate that *E. multilocularis* larvae are responsive to exogenously added host-insulin at physiological concentrations. First, 10 nM insulin significantly increased the production of metacestode vesicles from parasite stem cells as well as the re-differentiation of protoscoleces towards metacestode vesicles, and also significantly stimulated parasite stem cell proliferation in primary cell cultures and metacestode vesicles, as measured by the incorporation of BrdU. Second, the uptake of radioactively labelled glucose by metacestode vesicles was significantly stimulated in the presence of 10 nM host insulin. Third, exogenously added host insulin clearly affected the phosphorylation profiles of components of the PI3K/Akt signalling pathway in the metacestode. On the basis of these data, we propose that insulin constitutes an important host factor that influences the development and physiology of *E. multilocularis* larvae within the liver. The observed effects were most striking for initial metacestode development from stem cells, which could aid the parasite in establishing itself early during an infection, when it is most vulnerable to attacks by the host immune system [36]. Compared to primary cells, somewhat lower effects were observed on the proliferation of mature metacestode vesicles, which could be due to the fact that this stage contains significantly lower proportions of stem cells that are capable of proliferation than the primary culture system (Koziol *et al*., submitted for publication). On the other hand, given the important role of glycogen as the main energy source for larval cestode metabolism, the observed effects of host insulin on glucose uptake by *E. multilocularis* could be important for long-term persistence of the parasite within the host. Whether the insulin-stimulated re-differentiation of protoscoleces towards the metacestode is important *in vivo* still remains to be determined. Protoscolex re-differentiation in experimental secondary echinococcosis or following accidental or surgery-induced rupture of parasite cysts is a well described phenomenon [2,3] and at least for *E. granulosus* it is thought that parasite persistence within the host is aided by re-differentiation of existing protoscoleces once the mother hydatid cyst experienced physical damage [3]. In this regard, the influx of elevated concentrations of host insulin into ruptured parasite cysts, followed by increased re-differentiation of protoscoleces, may well contribute to prolonged parasite survival. However, whether these mechanisms are also relevant to *E. multiocularis* infections is still not clear. In any case, the observed effects of 1 nM and 10 nM insulin on protoscolex re-differentiation again demonstrate that *E. multilocularis* larvae are well responsive to physiological concentrations of insulin.

Since our data revealed that insulin significantly stimulates metacestode vesicle formation from primary cell cultures in a system that mimics the natural oncosphere-metacestode-transition, it is, of course, tempting to speculate that the relatively strict organ-tropism of *E. multilocularis* towards the host liver [1,2] may, at least in part, depend on the high insulin concentrations usually present in this organ. Although this is supported by our data showing that host insulin stimulates proliferation of *E. multilocularis* stem cells, which is in line with the role of insulin signalling in proliferation control of neoblasts in free-living flatworms [16], further experiments addressing insulin effects on naturally isolated oncospheres are necessary to obtain a conclusive picture. This would also require comparative analyses on oncospheres from *E. granulosus*, which display a relaxed liver organ tropism, and those of *Taenia solium* (or *Taenia saginata*), which usually don’t develop in the host liver, despite an entry route into the host comparable to that of *E. multilocularis*[2]. It is interesting to note in this context that Escobedo *et al*. [14] did not observe effects on *T. solium* cysticerci under high insulin treatment conditions that stimulated larval budding in *T. crassiceps*. However, care has to be taken in the interpretation of their results, since for *T. solium* the authors measured scolex evagination which is not, *per se*, a developmental process.

According to the theory of hormonal host-helminth cross-communication, endo- and paracrine hormonal systems of mammals (or even invertebrates) could influence the physiology and development of metazoan parasites through stimulation of evolutionarily conserved signalling systems [2,37-39]. This theory has thus far been supported by several *in vitro* studies showing that parasite surface receptor kinases of the insulin-, the EGF- and the TGF-β-families can principally bind respective host-derived hormones [10,11,13,37-41]. One of the most convincing examples supporting this theory has been brought up by Vicogne *et al*. [41] who demonstrated that human EGF can activate an EGF-receptor, such as tyrosine kinase of *S. mansoni in vitro* and at the surface of schistosomes, and that exogenously added EGF also influences protein and DNA synthesis in the parasite. We now propose the host-insulin-*E. multilocularis*-EmIR1 system as another example that supports this theory. Again, several lines of evidence clearly indicate that at least some of the effects of host insulin on *E. multilocularis* development and physiology involve binding of the host hormone to the insulin-receptor-like tyrosine kinase EmIR1. First, exogenously added host insulin influences EmIR1 phosphorylation patterns in the metacestode which is prevented in the presence of an anti-insulin-receptor inhibitor. Second, host insulin particularly influenced the phosphorylation of components of the PI3K/Akt pathway, which is known to act downstream of insulin-receptor tyrosine kinases in many organisms [7-9], and this was prevented in the presence of an insulin receptor inhibitor. Since the stimulation of the PI3K/Akt pathway through insulin receptors requires IRSs as intermediate signalling molecules [42], a binding site for which is present in EmIR1 (but not in EmIR2), the activation of this pathway in *E. multilocularis* most likely involves EmIR1. Third, although *E. multilocularis* encodes ILPs, the expression levels of the respective genes in the metacestode are very low and none of the parasite ILPs interacted with EmIR1 in yeast two-hybrid assays, indicating that host insulin is the only EmIR1 activating hormone present in significant concentrations around the growing metacestode. In this respect, it is even tempting to speculate that EmIR1 entirely lost the capacity to be stimulated by parasite-encoded ILPs since it is most active in parasite stages that have contact with elevated concentrations of host insulin. We, thus, propose that several of the actions of insulin on the *E. multilocularis* metacestode, particularly the stimulation of glucose uptake and the stimulation of metacestode proliferation, are mediated by direct binding of the host hormone to EmIR1, followed by subsequent activation of insulin-dependent parasite signalling pathways. This should be particularly relevant in the *Echinococcus* GSCs, which display the highest expression levels of EmIR1 and are the cell type responsible for carbohydrate storage.

Although EmIR1 at the protein level was not detected in the *E. multilocularis* primary cell cultivation system, we could observe clear effects of host insulin on the formation of metacestode vesicles from parasite stem cells. These effects are, thus, most probably mediated independently of EmIR1 and in the present study we identified a second *E. multilocularis* insulin receptor molecule, EmIR2, which could be involved in the effects on parasite stem cells. On the one hand, our histochemical analyses showed that EmIR2 expression is dispersed through primary cell aggregates, which contain a large number of parasite stem cells [19]. Furthermore, the *in situ* hybridization experiments presented in this work clearly indicate that at least in developing protoscoleces, *emir2* transcripts are closely associated with the proliferation zone where parasite stem cells are most active (Koziol *et al*., submitted for publication), indicating a link between EmIR2 and stem cell proliferation or differentiation. The presence of two insulin receptor encoding genes in *E. multilocularis* closely resembles the situation in the related schistosomes, which also express two molecules of this class [11-13]. As with the schistosome receptor LBDs, which interacted with human insulin in the yeast two-hybrid system [11,13], we herein demonstrated that in addition to EmIR1, EmIR2 can also interact with the host hormone. Since the *Echinococcus emilp2* gene was expressed at low, but detectable, levels in primary cells and since the encoded peptide, EmILP2, interacted with EmIR2 in the yeast two-hybrid system, we cannot exclude that a certain level of stimulation of EmIR2 by EmILP2 in primary cells could contribute to initial parasite development within the liver. However, our experiments clearly indicate that physiological levels of human insulin, that should be present at the site of initial parasite development from the oncosphere, can significantly add to these effects. Hence, it is conceivable that during the oncosphere-metacestode transition both EmILP2 and human insulin bind to EmIR2, which could lead to higher activation of the parasite receptor than through EmILP2 alone, and which could thus promote rapid parasite establishment. Whether this indeed occurs *in vivo* and which parasite signalling pathways act downstream of EmIR2, given that it lacks the conserved NPXY motif, still remains to be established. Unfortunately, and in contrast to the metacestode vesicle culture system, membrane fractionation and insulin stimulation studies are very difficult to carry out on the stem cell cultivation system due to the fragility of stem cell aggregates and their high sensitivity to serum-free cultivation conditions. Nevertheless, given that EmIR2 is capable of interacting with human insulin in the yeast two hybrid system and that it is expressed as the only parasite insulin receptor in the primary cell system, hormonal host-parasite cross-communication through insulin-binding to EmIR2 could indeed play a significant role in parasite establishment within the liver.

Ahier *et al*. [11] and You *et al*. [13] previously used inhibitors specifically designed to bind to insulin receptor-like kinases and observed deleterious effects on the uptake and consumption of glucose by schistosomes, indicating that at least the mechanisms of glucose uptake, similar to *Echinococcus* as shown in this study, are under the control of insulin signalling in these parasites. In the present study, we employed HNMPA(AM)_3_, the same inhibitor used by You *et al*. [13], and observed various effects on the development of metacestode vesicles from primary cells, on the survival of mature metacestode vesicles and on the re-differentiation process from protoscoleces towards the metacestode. In mature metacestode vesicles, only relatively high concentrations (100 μM) of HNMPA(AM)_3_ led to killing and we suggest that this mostly involved binding of the drug to EmIR1, accompanied by defects in glucose uptake and consumption. That the drug can principally bind to EmIR1 is supported by our *in silico* analyses showing that the parasite receptor’s ATP-binding pocket is capable of harbouring HNMPA(AM)_3_ with considerable affinity. Compared to mature metacestode vesicles, the effects of HNMPA(AM)_3_ on primary cells were much more dramatic. Already at a concentration of 25 μM, the insulin receptor inhibitor completely prevented the formation of metacestode vesicles from parasite stem cells. Since EmIR1 is not expressed in this parasite stage, we suggest that EmIR2 is also capable of binding HNMPA(AM)_3_, maybe even with higher affinity than EmIR1. Indeed, in a recent report Vanderstraete *et al*. [31] demonstrated that HNMPA(AM)3 inhibits the schistosome receptor SmIR1 (which is the ortholog to EmIR2) with much higher efficacy (10 to 100 fold more) than SmIR2 (ortholog to EmIR1). When applied to the *Echinococcus* system, this could explain the relative resistance of the (EmIR1 expressing) metacestode to the drug when compared to the (EmIR2 expressing) primary cell system. However, care has to be taken in the interpretation of data on insulin inhibitor effects on flatworms since Vanderstraete *et al*. [31] also showed that these can affect a structurally diverse family of receptor kinases that are composed of an extracellular Venus FlyTrap (VFT) motif and an intracellular, insulin receptor-like TKD. In *S. mansoni*, two of these kinases, named SmVKR1 and SmVKR2, are expressed and a panel of available insulin receptor inhibitors that showed effects on SmIR1 and SmIR2 also affected SmVKR1 and SmVKR2 in a similar manner [31]. In the *E. multilocularis* genome, only one gene encoding such a tyrosine kinase, EmVKR, is present and transcriptome data indicate that it is expressed in a similar manner as *emir2* (data not shown). For the inhibitor data concerning EmIR1 phosphorylation upon addition of insulin, we do not see interpretation problems since this was carried out specifically for EmIR1, immunoprecipitated from membrane fractions. However, at least some of the effects we observed on entire *Echinococcus* larvae after application of HNMPA(AM)_3_ could indeed be due to inhibition of EmVKR rather than EmIR1 and EmIR2. Unfortunately, it is presently not possible to clearly distinguish between these possibilities since highly selective inhibitors for the parasite insulin receptors versus the VKR receptors are not available [31] and since RNAi methodology for *E. multilocularis* is still in its infancy. Nevertheless, our data indicate that the insulin signalling system of *E. multilocularis*, including insulin receptor kinases, EmVKR, and downstream signalling components, might be a fruitful target for the development of novel chemotherapeutics, as has previously been argued in the case of schistosomes [11-13,31].

In summary, our data indicate an important role of host insulin on the development of *E. multilocularis* larvae within the host’s liver. We also showed that this involves hormonal host-parasite cross-communication via evolutionarily conserved signalling systems, which is particularly striking for EmIR1 concerning glucose uptake in GSCs of the metacestode and, most likely, also applies to EmIR2 in the primary cell system. Using a well-known inhibitor of insulin receptor signalling, we also demonstrated clear effects on parasite survival and, particularly, development. Although HNMPA(AM)_3_ might not be as efficient as other kinase inhibitors, such as pyridinyl imidazoles [43] or imatinib [44], in inducing killing of the metacestode, which is the main target of chemotherapy against alveolar echinococcosis, our study now opens the way for the development of more specific inhibitors that could be used to affect glucose uptake by the parasite during development. Furthermore, due to their obvious effects on parasite stem cell proliferation, insulin receptor inhibitors might be used to inhibit asexual multiplication of an already established parasite mass or to prevent metastasis formation from stem cells in advanced cases of the disease [2]. Since the somewhat lower efficacy of HNMPA(AM)_3_ to inactivate metacestode vesicles (when compared to primary cells) could at least in part be due to problems in penetrating the laminated layer which surrounds the parasite cells, issues of improved tissue penetration should also be considered in studies on the development of anti-insulin signalling drugs against AE.

## Conclusions

The *E. multilocularis* metacestode larval stage displays a marked organ-tropism towards the mammalian host’s liver where it grows infiltratively, like a malignant tumour, and where the highest concentrations of insulin within the mammalian body can be found. We herein demonstrate that mammalian insulin influences *E. multilocularis* larval development at physiological concentrations which, to the best of our knowledge, is also the first report on stimulatory effects of physiological insulin concentrations on any flatworm parasite. Our data indicate that *E. multilocularis* insulin signalling pathways, consisting of two insulin receptor-like tyrosine kinases and downstream components of the PI3K/Akt-pathway, are mediating these effects, which supports the theory that hormonal host-parasite cross-communication through evolutionarily conserved signalling systems plays an important role in *Echinococcus* infections. That the effects we observed *in vitro* are also of relevance *in vivo* is indicated by the fact that the metacestode stage, which grows continuously within the host liver, is not producing intrinsic insulin-like peptides for the main receptor of this stage, EmIR1, thus leaving host-derived insulin as the only relevant hormone of this class at the site of infection. Although further investigations are needed to establish a clear connection between the parasite’s insulin responsiveness and the marked organ-tropism towards the host liver, we nevertheless suggest that the constantly elevated supply of insulin within the liver (when compared to serum or other organs) at least contributes to the initial development of the metacestode from parasite stem cells, and supports asexual multiplication of the metacestode. By our investigations on the inhibition of insulin signalling pathways in *E. multilocularis*, we also identified a lead compound that could facilitate the development of novel and effective anti-echinococcosis drugs in the future. Investigations into this direction, addressing the parasite’s insulin receptor-like kinases, but also downstream components such as PI3K and Akt, are currently underway.

## Methods

### Organisms and culture methods

Experiments were performed with the *E. multilocularis* isolates H95 [32] and JAVA [45] which were continuously passaged in mongolian jirds (*Meriones unguiculatus*) as previously described [18]. Since we observed an influence of the period of intraperitoneal jird passages on the reproducibility of the experiments (that is, ‘younger’ isolates were consistently more responsive to insulin than ‘old’ isolates), we always used the most recent isolate that was available in the laboratory for the experiments. *In vitro* cultivation of metacestode vesicles under axenic conditions as well as the isolation and cultivation of primary cell cultures was carried out as previously described [18,19]. Protoscoleces were isolated from *in vivo* cultivated parasite material according to a previously established protocol [46] and were activated by pepsin/low pH treatment (mimicking the transition to the definitive host) as previously described [33]. Life-dead staining of protoscoleces was carried out by incubation of protoscoleces with 0.03% methylene blue for one minute.

### Insulin and inhibitor treatment of parasite larvae

Metacestode vesicles (JAVA) of a diameter of 3 to 4 mm were manually picked from axenic culture [19], washed in PBS and incubated in 12-well plates in the presence of conditioned medium [18]. Viability and integrity of the vesicles were measured microscopically after incubation for seven days in the presence or absence of the insulin receptor inhibitor HNMPA(AM)_3_ (Enzo Life Sciences, Lörrach, Germany). Primary cells were isolated from six- month-old axenic vesicles [18] and incubated in conditioned medium supplemented with recombinant human insulin (Sigma-Aldrich, Hamburg, Germany), DMSO and HNMPA(AM)_3_. Primary cell incubation was carried out for seven days in the case of haematoxylin staining of sections. Metacestode vesicle formation from parasite stem cells was measured after three weeks of incubation by counting free swimming, intact vesicles and microscopic measurement of the size and amount of primary cell aggregates [43,44]. Protoscoleces were incubated in hepatocyte-conditioned medium supplemented with insulin for three weeks (in insulin stimulation experiments) or DMSO and HNMPA(AM)_3_ for two weeks (in inhibitor experiments). Re-differentiation was evaluated by counting vesicular protoscoleces. Protoscolex viability was measured by staining with 0.03% methylene blue for one minute. All experiments were carried out independently at least three times.

### BrdU uptake assays

Metacestode vesicles (3 to 4 mm; JAVA) were manually picked from axenic cultures [19], washed in PBS and incubated in 12-well plates in the presence of hepatocyte-conditioned medium supplemented with insulin and 1 mM BrdU for two days. Chromosomal DNA was subsequently isolated and 500 ng DNA was coated onto an ELISA plate using DNA coating solution (Thermo Scientific, Bonn, Germany) according to the product manual. BrdU incorporation was detected using the colorimetric BrdU ELISA kit (Roche, Mannheim, Germany). Stimulation of freshly isolated primary cells was carried out for 24 hours, followed by 4 hours of incubation with 1 mM BrdU in a 96-well plate. For the BrdU-ELISA the colorimetric BrdU ELISA kit (Roche) was used. The lysed cells were blocked with 2% skim milk in PBS for one hour.

### Glucose uptake assay

Metacestode vesicles (JAVA) were manually picked from *in vitro* cultures, washed in PBS and incubated overnight in (D)MEM supplemented with 0.2% FCS and 2.5 mM glucose. Medium was changed and supplemented with 0.1 μCi [^14^C]-D-glucose (Hartmann Analytic, Braunschweig, Germany) to which either 10 nM human insulin or 10 nM insulin plus 100 nM Na_3_VO_4_ were added. Samples were incubated for one hour at 37°C and then washed in PBS. Vesicles were lysed with 0.15 M NaOH for five minutes at room temperature and centrifuged for one minute at 2,000 rpm. The supernatant was resuspended in UltimaGold (Perkin-Elmer, Rodgau-Juegesheim, Germany) and radioactivity was measured in a liquid scintillation counter. Data were expressed relative to control sample radioactivity.

### Nucleic acid isolation, cloning and sequencing

RNA isolation from *in vitro* cultivated axenic metacestode vesicles (H95, JAVA), protoscoleces (JAVA) and primary cells (one week; JAVA) was performed using a Trizol (5Prime, Hamburg, Germany)-based method as previously described [43]. For reverse transcription, 2 μg total RNA was used and cDNA synthesis was performed using oligonucleotide CD3-RT [46]. PCR products were cloned using the PCR Cloning Kit (QIAGEN, Hilden, Germany) and sequenced employing an ABI prism 377 DNA sequencer (Perkin-Elmer). For cloning and sequencing the *emir2* cDNA, available genomic sequences for *E. multilocularis* were used [22]. After partial amplification of three overlapping fragments (encoding the LBD, the intracellular portion, and the region between LBD and the transmembrane domain), cloning and sequencing, which largely confirmed the sequence as presented in GeneDB [47], the full length cDNA was amplified from metacestode mRNA preparations using the primers EmIRb-F1-dwHindIII (5’-GCT CGC AAG CTT ACA GAC AAT GAA TGT GC-3’) and EmIRb-up-KpnI (5’-CCT CAG GTA CCC CAT GTG AGA GAG TGG AAG TTC-3’), cloned into pSecTag2/Hydro (Life Technologies, Darmstadt, Germany), sequenced again and was used for all subsequent amplification steps. Likewise, the *emilp1* and *emilp2* cDNAs were full-length amplified from protoscolex mRNA preparations using primers ilp1HindIIIdw (5’-GCA TAA GCT TGT CGC CTC TGG CCC AAG-3’) and ilp1NotIStop (5’-GCA TGC GGC CGC TCA GCC TTT TGC ACA-3’) as well as ilp2HindIIIdw (5’-GCA TAA GCT TGG TAT CAC CTC TTC AT-3’) and ilp2NotIStop (5’-GCA TGC GGC CGC CTA AAC AAC AGC ATT-3’), respectively, and cloned as described above before sequencing. All sequences as determined in this study have been deposited at the EMBL Nucleotide Sequence Database under the accession numbers [EMBL:HG326255] (*emir2*), [EMBL:HG326256] (*emilp1*), [EMBL:HG326257] (*emilp2*), [EMBL:HF934007] (*emakt*), and [EMBL:HF934006] (*em4ebp*).

### RT-PCR analysis

Total RNA was isolated from axenically cultivated metacestode vesicles (JAVA), primary cell cultures as well as non-activated and activated protoscoleces and cDNA was produced as described previously [43]. Ten-fold, serial dilutions of normalized cDNA were then used as template for PCRs using intron-flanking, *emir1*-specific primers RC19 (5′- GAT GAT TCC TTC GAT TTG CA- 3′) and RC21 (5′- TAA ACG AGA CGT TCC CAA CAT G - 3′) as well as the intron-flanking, *emir2*-specific primers EmIRbdw3 (5’-GGA CGA GTG GGA GGT GG-3’) and EmIRbup3 (5’-GAA CTG TTT CAT GTG GGA GG-3’). The PCR program was 94°C for one minute, 59°C for 30 seconds and 72°C for 30 seconds at 35 cycles. The constitutively expressed control gene *elp*[46] was amplified using intron-flanking primers Em10-15 (5′- AAT AAG GTC AGG GTG ACT AC −3′) and Em10-16 (5′- TTG CTG GTA ATC AGT CGA TC −3′) using the PCR program 94°C for one minute, 53°C for 30 seconds and 72°C for 30 seconds at 35 cycles. PCR products were separated on a 1% agarose gel and stained with ethidium bromide.

### Generation of anti-EmIR1 and anti-EmIR2 immune sera

Antibodies were raised against the intracellular domain of EmIR1 (M1129- C1749) [10]. The respective cDNA regions were amplified using primers CK5 (5′- ATC GCT GGA TCC ATA CAT CGC ATT CGA AAG AA −3′) and CK6 (5′- AAC ACA AGA TCT TGA ACA AGA CGA CCC ATC ACC GTC A −3′). The PCR product was ligated into the pBAD/Thio-Topo® vector (pBAD⁄TOPO® ThioFusion™ Expression Kit, Invitrogen, Karlsruhe, Germany) and expressed and purified according to the manufacturer’s instructions. Immunisation of a rabbit with the purified protein was performed by Immunoglobe (Himmelstadt, Germany) using program PRO-10 W-STD [48]. Likewise, nt sequences encoding the intracellular region of EmIR2 were amplified using primers emirbF3dw (5’-GCA ACC ACC TTC GCT AAT G-3’) and emir2-intra-up (5’-CTC AGA ATT CAT GTG AGA GTG GAA G-3’) and cloned into the pBAD/TOPO ThioFusion expression plasmid (Invitrogen). The EmIR2-Thio construct was expressed in *Escherichia coli* as described above for EmIR1 and the protein was purified via the His-tag. Elution fractions were dialysed and used for rabbit immunisation according to the procedure described above. In subsequent western blot analyses the purified immune-serum only detected EmIR2-Thio and EmIR2-GST, but not EmIR1-GST [see Additional file 4], thus confirming specificity.

### SDS -PAGE and Western Blot analysis

Lysates of axenically cultivated metacestode vesicles (JAVA, H95) were obtained by mechanically disrupting the cysts and centrifugation for five minutes at 800 g and 4°C. The pellet was then lysed with lysis buffer (20 mM Tris–HCl, pH 8.0; 150 mM NaCl; 1 mM ethylenediaminetetraacetic acid (EDTA) pH 8.0; 1% Triton X-100; 2% sodium deoxycholate; 1 mM Na_3_VO_4_; 10 mM NaF) supplemented with 1× protease inhibitor (Complete Protease Inhibitor Cocktail Tablets, Roche) for one to two hours at 4°C under constant rotation. The protein concentration of the samples was determined and equal amounts of protein were loaded onto a 10% SDS gel. Primary cells, non-activated and activated protoscoleces were centrifuged for five minutes at 800 g and 4°C and lysed with lysis buffer for one to two hours at 4°C under constant rotation. Proteins were subsequently separated (10% SDS-PAGE), transferred to a membrane and detected with antibodies. The following antibodies were used: anti-EmIR1 (1:1,000), anti-EmIR2 (1:1,000), and anti-rabbit immunoglobulin G-horseradish peroxidase (IgG-HRP) (1:5,000; Jackson, Immuno Research, West Grove, PA, USA) as secondary antibody. For the β-actin control, a rabbit anti-β-actin antibody (Cell Signalling, Frankfurt/Main, Germany; 1:1,000) was used.

### Immunohistochemistry and electron microscopy

For conventional transmission electron microscopy (TEM) and increased preservation of carbohydrate based structures, such as glycogen, *in vitro* cultured *E. multilocularis* metacestodes were fixed in 100 mM sodium cacodylate buffer, pH 6.8, containing 2.5% glutaraldehyde and 0.1% tannic acid for four hours at room temperature [49]. After three washes in cacodylate buffer and post-fixation in 2% osmium tetroxide in cacodylate buffer for two hours at room temperature, specimens were pre-stained in 1% uranyle acetate for 30 minutes at room temperature. After washing in water, samples were dehydrated in a stepwise gradient of ethanol (30, 50, 70, 90, 3×100%) and were embedded in Epon 812 epoxy resin (Sigma), with three changes of resin within 48 hours. Blocks were polymerized at 60°C for 24 hours.

Immunofluorescence and immunogold-TEM employing the anti-EmIR1 antiserum or a general anti-*Echinococcus* metacestode antigen antibody [50] were done on sections obtained from metacestodes embedded in acrylic LR-White resin (Sigma). To this end, *in vitro* cultured metacestodes were washed twice with PBS and then placed in fixation solution (3% paraformaldehyde/0.05% glutaraldehyde in 100 mM sodium cacodylate buffer (pH 6.8)) for 30 minutes at room temperature, washed in sodium cacodylate buffer and placed into 20 mM glycine in PBS for 30 minutes on ice. They were dehydrated in steps of 30, 50, 70, 90 and 100% ethanol on ice, followed by two changes of LR-White resin. Specimens were infiltrated at −20°C for 24 hours and, subsequently, the resin was changed again. Polymerization of the resin was done at 60°C for 24 hours under exclusion of O_2_ and tissue blocks were stored at 4°C until use.

Sections of 1 μm thickness for immunofluorescence and 80 nm thickness for conventional or immunogold TEM were cut on a Reichert and Jung ultramicrotome (Vienna, Austria). For immunofluorescence, sections were mounted onto poly-l-Lysine coated glass coverslips. For TEM, sections were placed onto formvar-carbon coated grids.

For immunofluorescence staining, glass coverslips were placed into blocking buffer (PBS, 3% BSA) for one hour and were then incubated with anti-EmIR1 antiserum, diluted 1:250 in PBS, 0.3% BSA for one hour at room temperature. Secondary antibodies were goat anti-rabbit FITC (fluorescein isothiocyanate; Sigma, Buchs, Switzerland) diluted 1:200 and applied for 30 minutes. The coverslips were then finally washed for 10 minutes in PBS and were mounted on glass slides with VECTASHIELD® Mounting Medium containing DAPI (4′,6-diamidino-2-phenylindol; Vector Laboratories, Burlingame, CA, USA). Slides were observed on a Nikon Eclipse® E800i digital confocal fluorescence microscope (Nikon Precision Inc. Belmont, CA, USA) and processing of images was performed using the Openlab 2.0.7 software (Improvision, Waltham, MA, USA).

For immunogold TEM, grids were placed onto drops of blocking solution for one hour, and were then incubated on anti-EmIR1 antiserum as for immunofluorescence. Secondary antibody-gold conjugates were 10 nm-gold-goat anti-rabbit conjugates (Aurion, Wageningen, NL), diluted 1:10 in PBS, 0.3% BSA, and were applied for one hour, followed by three washes in PBS, five minutes each. Grids were briefly dipped into distilled water and air dried. Contrasting of both conventional and immunogold TEM samples was done with uranyle acetate and lead citrate. Specimens were viewed on a Phillips 400 TEM operating at 80 kV.

For immune-histochemistry using the anti-EmIR2 antiserum, samples were embedded in Technovit 8100 (Heraeus Kulzer, Wehrheim, Germany) and 4 μm sections were taken on glass slides. Sections were dried for two hours at 37°C and cauterise for four minutes with acetone. Rehydration was performed by incubation for five minutes in 100% ethanol, five minutes in 96% ethanol, five minutes in 70% ethanol and five minutes in 1x PBS. Samples were then permeabilised for seven minutes with 1% Triton X-100 in PBS and rinsed three times with PBS. For blocking endogenous peroxidases, slides were incubated for 10 minutes with 0.3% H_2_O_2_ in methanol and washed two times for 10 minutes with PBS. The first antibody (anti-EmIR2, 1:10 in blocking buffer) was added and incubation was performed overnight at 4°C (humid chamber). Samples were then washed three times for five minutes with PBS and the second antibody (POX-anti-rabbit, 1:50 in blocking buffer) was incubated for three hours at room temperature in a humid chamber. Samples were washed again, substrate solution (2 mg diaminobenzidine (DAB); 2 ml PBS; 2 ml H_2_0; 1.34 μl 30% H_2_O_2_) was added and incubated at room temperature until the reaction was stopped by rinsing in H_2_0. Counterstaining with haemotoxylin was carried out by incubation for six minutes with Haemalaun (Roche). Slides were washed and passed through an increasing ethanol series (five minutes in 70% ethanol, five minutes in 96% ethanol, five minutes in 100% ethanol; five minutes in xylol) and mounted with Entellan (Merck, Darmstadt, Germany). As a control, staining of parasite sections with only the secondary antibody (POX-anti-rabbit) was performed and continuously yielded negative results.

### *In vitro* phosphorylation of parasite proteins

#### EmIR1 phosphorylation in membrane fractions

An assay adopted from Vicogne *et al*. [41] was used. *In vitro* cultivated metacestode vesicles (H95) were isolated and incubated in (D)MEM 0.2% FCS (supplemented with 10 U/ml penicillin G/streptomycin, 100 μM L-cysteine, 10 μM bathocuproinedisulphonic acid, 0.01% β-mercaptoethanol) for 24 hours at 37°C and 5% CO_2_. After this incubation period, vesicles were washed (1 x PBS) and transferred into a 15 ml Falcon tube. Excess PBS was removed and 0.5 ml homogenization buffer (20 mM Tris–HCl, pH 7.5; 150 mM NaCl; 1 mM EDTA pH 8.0; 1% Triton X-100; 1 mM Na_3_VO_4_; 10 mM NaF; 1 mM phenylmethanesulphonyl fluoride (PMSF); 10 μg/ml apronitin A; 1 μg/ml Pepstatin A; 1 μM leupeptin hemisulphate) were added per 1 ml intact vesicles. The vesicles were mechanically homogenized at 4°C before the membrane fraction was pelleted by centrifugation (3,000 rpm, three minutes) at 4°C. Supernatant was discarded and the pellet was resuspended in fresh homogenization buffer. The suspension was aliquoted into reaction tubes and either human insulin (Hoechst; 100 nM – saturating concentration to ensure maximal activation [25-30]) or human IGF (Immunotools, Friesoythe, Germany; 100 nM) were added. After 10 minutes at 37°C, the membrane fraction was pelleted again (3,000 rpm, three minutes at room temperature) and resuspended in 300 μl kinase buffer (50 mM Tris–HCl pH 7.5; 2 mM MnCl_2_; 15 mM MgCl_2_; 0.1% Triton X-100; 1 mM Na_3_VO_4_; 10 mM NaF; 1 mM PMSF; 10 μg/ml apronitin; 1 μg/ml Pepstatin A, 1 μM leupeptin hemisulphate) containing 100 μM HNMPA(AM)_3_ or an equal volume of DMSO. After 30 minutes at 37°C, the kinase buffer was supplemented with 50 μM [^32^P] γ-ATP (4.4 ml of 110 TBq/mmol) and phosphorylation was carried out for 40 minutes at 30°C. The membrane fraction was then briefly centrifuged (five minutes, 1,300 rpm at room temperature), the supernatant was discarded, and the pellet was resuspended in 1 ml lysis buffer (20 mM Tris–HCl pH 8.0; 150 mM NaCl; 1 mM EDTA pH 8.0; 1% Triton X-100; 2% sodium deoxycholate; 1 mM Na_3_VO_4_; 10 mM NaF; 1 mM PMSF; 10 μg/ml apronitin; 1 μg/ml Pepstatin A; 1 μM leupeptin hemisulphate). To solubilise membrane bound proteins, samples were gently agitated at 4°C for one hour. Insoluble material was removed by centrifugation (five minutes, 1,300 rpm at room temperature) and the EmIR1 β-subunit was immunoprecipitated from the supernatant using the anti-EmIR1 antiserum (1:100) employing agarose G-beads (Upstate, Lake Placid, NY, USA) according to the manufacturer’s instructions. Phosphorylation of immunoprecipitated proteins was subsequently analysed by SDS-PAGE (8% polyacrylamide (PAA)), followed by transfer onto a nitrocellulose membrane and autoradiography (X-ray film; Fuji).

#### EmIR1 phosphorylation in intact vesicles

Intact *in vitro* cultivated metacestode vesicles (H95) were manually picked, transferred into Falcon tubes and incubated in (D)MEM (0.2% FCS) in the presence or absence of 100 nM insulin (saturating concentration to ensure maximal activation [25-30]). After 10 minutes incubation, medium was removed and the metacestode vesicles were mechanically disrupted and pelleted by centrifugation (one minute, 1,300 rpm, 4°C). The pellet was resuspended in 1 ml lysis buffer (see above), agitated for one hour at 4°C and insoluble material was removed by centrifugation (15 minutes, 1,300 rpm, 4°C). Immunoprecipitation of EmIR1 using the anti-EmIR1 antiserum was carried out as described above and precipitated proteins were analysed by Western blotting with the anti-EmIR1 antiserum. Tyrosine phosphorylation of the EmIR1 β-subunit was carried out using an anti-phospho-tyrosine antibody (P-Tyr 100, Cell Signalling).

#### Phosphorylation of components of the PI3K/Akt paythway

Intact *in vitro* cultivated metacestode vesicles (JAVA) were incubated for 16 hours in (D)MEM (0.2% FCS), followed by stimulation with 10 nM insulin for 5, 30 and 60 minutes. In some experiments, HNMPA(AM)_3_ or the PI3K inhibitor LY294002 [51] (both at 100 μM) were added two hours prior to insulin stimulation. Samples were then put on ice and washed once with cold PBS supplemented with 1 mM Na_3_VO_4_ and 10 mM NaF. Vesicles were then mechanically disrupted and hydatid fluid was removed after centrifugation (three minutes, 800 g, 4°C). Crude lysates were then produced by adding 5 x sample buffer (15% Tris–HCl pH 6.8; 50% glycerol; 10% SDS; 25% β-meracptoethanol) to a final concentration of 1x. Samples were then boiled for 10 minutes and centrifuged for one minute at 11,000 g. The supernatant was separated by SDS-PAGE and Western blot analysis was carried out using the following antibodies: anti-phospho-4E-BP1 (T37/46) (Cell Signaling, Frankfurt, Germany), and anti-phospho-Akt Substrate (RXRXXS/T) (Cell Signaling). For secondary antibodies anti-mouse IgG-HRP (1:10000; Jackson, Immuno Research) and anti-rabbit IgG-HRP (1:5000; Jackson, Immuno Research) were used.

### Yeast two-hybrid analyses

The Gal4-based MATCHMAKER system (Clontech, Mountain View, CA, USA) was used essentially as described previously [10,11,13]. Constructs for the fusion of the EmIR1- and HIR- LBDs to the Gal4 activation domain (AD) as well as human pro-insulin to the Gal4 DNA binding domain (BD) have been described previously [10]. For fusing the EmIR2 LBD with the Gal4 AD, the respective cDNA sequences were amplified using primers emir2ex-EcoRI (5’-GTC ACG AAT TCA CAG ACA ATG AAT GTG C-3’) and emir2ex-XhoI (5’-GTT GAC TCG AGG TCC TTC ACA GAA GC-3’) and were cloned into vector pGADT7 using restriction sites incorporated into the primer sequences. For fusions of the *Echinococcus* ILPs with the Gal4 BD, corresponding cDNA sequences were amplified using primers emilp1EcoRI (5’-GTC ACG AAT TCT TTG AGA TGG ATA AAA CG-3’) and emilp1BamHI (5’-GCG ATG GAT CCG CCT TTT GCA CAG AAC-3’) (for *emilp1*) as well as emilp2EcoRI (5’-GTC ACG AAT TCG TAT CAC CTC TTC ATG-3’) and emilp2BamHI (5’-GCG ATG GAT CCA ACA ACA GCA TTG AG-3’) (for *emilp2*) and cloned into plasmid pGBKT7 via restriction sites incorporated into the primer sequences. All constructs were checked by sequencing for correct reading frames. Co-transformation of the plasmid constructs into yeast and growth analysis was performed essentially as previously described [10]. As controls, empty vectors and fusion proteins with the *E. multilocularis* protein Elp (Ezrin-radixin-Moesin-like protein [52]) were used as previously described [11].

### EdU labeling and detection

A total of 50 μM EdU (Life Technologies) was added to metacestode *in vitro* cultures (JAVA) and incubated for five hours. For fixation, metacestode vesicles were gently opened using a syringe tip to allow entry of the fixative and detection reagents. The samples were fixed for one hour at room temperature in 4% paraformaldehyde prepared in PBS (PFA-PBS). Detection was performed with the Click-iT® EdU Alexa Fluor® 555 Imaging Kit (Abcam, Cambridge, UK) as described by the manufacturer for sections, but with a modified protocol in which all steps were doubled in length and the washes were increased in number. EdU detection was performed after carrying out the *in situ* hybridization protocol. Finally, samples were co-stained with DAPI.

### Whole-mount *in situ* hybridization (WMISH)

Digoxigenin-labeled probes were synthesised by *in vitro* transcription with T7 and SP6 polymerases (New England Biolabs, Frankfurt/Main, Germany), using the DIG RNA labeling mix (Roche, Mannheim, Germany) as described by the manufacturer from an *emir2* cDNA fragment cloned into pDrive using the Qiagen PCR cloning system. Primers used for amplification of the probe were emir2-dw2 (5’-ACA GAC AAT GAA TGT GCT TCC C-3’) and emir2-up5 (5’-CCA TTC GTA AAA ACC AGC GC-3’), which encompass an approximately 3 kb fragment of the *emir2* cDNA region encoding the extracellular portion of the receptor.

The WMISH protocol was adapted from methodology described in [53] and a detailed protocol will be published elsewhere (Koziol *et al*., submitted for publication).

### Computer analyses

Amino acid comparisons were performed using BLAST on the nr-aa database collection available under [54]. Genomic analyses and BLAST searches against the final assembly version of the *E. multilocularis* genome [22] were done using the respective resources of the Sanger Institute (Hinxton, UK) [47].

### Ethical approval

All experiments were carried out in accordance with European and German regulations on the protection of animals (*Tierschutzgesetz*). Ethical approval of the study was obtained from the local ethics committee of the government of Lower Franconia (55.2-2531.01-31/10).

## Abbreviations

4E-BP: eukaryotic translation initiation factor 4E-binding protein; AD: activation domain; AE: alveolar echinococcosis; BD: binding domain; BLAST: Basic Local Alignment Search Tool; BrdU: bromodeoxyuridine; BSA: bovine serum albumin; CDD: conserved domain database; DAPI: 4',6-diamidino-2-phenylindole; (D)MEM: (Dulbecco’s) modified Eagle’s medium; DMSO: dimethylsulphoxide; EDTA: ethylenediaminetetraacetic acid; EdU: 5-ethylnyl-2’-deoxyuridine; EGF: epidermal growth factor; ELISA: enzyme-linked immuosorbent assay; ERK: extracellular signal regulated kinase; FCS: fetal calf serum; GSC: glycogen storage cell; HIR: human insulin receptor; HNMPA: 2-hydroxynaphthalen-1-yl-methylphosphonic acid; HRP: horseradish peroxidase; IGF: insulin-like growth factor; IgG: immunoglobulin G; ILP: insulin-like peptide; IRS: insulin receptor substrate; LBD: ligand binding domain; MAPK: mitogen-activated protein kinase; PBS: phosphate-buffered saline; PI3K: phosphatidyl-inositol-3-phosphate kinase; PKB: protein kinase B; PMSF: phenylmethanesulphonyl fluoride; SMART: Simple Modular Architecture Research Tool; TEM: transmission electron microscopy; TGF: transforming growth factor; TKD: tyrosine kinase domain; WMISH: whole mount *in situ* hybridization.

## Competing interests

The authors declare that they have no competing interests.

## Authors’ contributions

The study was conceived by KB with contributions from TD and AH. The experiments were designed by KB, TD, and AH. The experiments were performed and analysed by SH, CK, UK, DS, MS, SF, BS and VG. Method development was carried out by UK, SH, CK, DS, MS, and AH. The manuscript was written by KB with contributions from TD, AH and UK. All authors have read and approved the final manuscript.

## Supplementary Material

Additional file 1 **The *Echinococcus multilocularis *** **life cycle.** Schematic representation of the *E. multilocularis* life cycle and suggested actions of host insulin on parasite development/physiology. Click here for file

Additional file 2 **Amino acid sequence comparisons between *Echinococcus* and human insulin receptors.** Pileups of LBDs and TKDs of insulin receptors from *Echinococcus* and human origin. Click here for file

Additional file 3 **Gene expression profiles of *Echinococcus* insulin signalling components during the life cycle.** Diagram displaying transcriptome data concerning the expression of *emir1*, *emir2*, *emilp1*, and *emilp2* in larval and adult stages. Click here for file

Additional file 4 **Testing the anti-EmIR1 and anti-EmIR2 antisera.** File showing Western blot and immunoprecipitation analyses concerning EmIR1 and EmIR2 using the generated antisera. Click here for file

Additional file 5 ***In silico* analyses.** Figure showing *in silico* models for the binding of HNMPA(AM)3 to the TKD of EmIR1. Click here for file

Additional file 6 **Components of the *E. multilocularis* insulin signalling pathways.** Table with *E. multilocularis* genes predicted to be involved in insulin signalling pathways according to the *E. multilocularis* genome sequencing project. Click here for file

Additional file 7 **Amino acid sequence comparison of PKB components from different organisms.** File showing sequence alignment of the *E. multilocularis* PKB-homolog EmAkt with PKBs from human and *Drosophila* origin. Click here for file

Additional file 8 **Amino acid sequence comparison of 4E-BP orthologs from different origin.** File showing sequence alignment of the *E. multilocularis* 4E-BP ortholog with those of human and insect origin. Click here for file
