# Supplementary material for: Host insulin stimulates Echinococcus multilocularis insulin signalling pathways and larval development
Source: BMC Biol. 2014 Jan 27;12:5. doi: 10.1186/1741-7007-12-5 (PMC3923246; doi:10.1186/1741-7007-12-5)
Supplement: Additional file 1 — The Echinococcus multilocularis life cycle. Schematic representation of the E. multilocularis life cycle and suggested actions of host insulin on parasite development/physiology. [file 1741-7007-12-5-S1.pdf]

Additional file 1

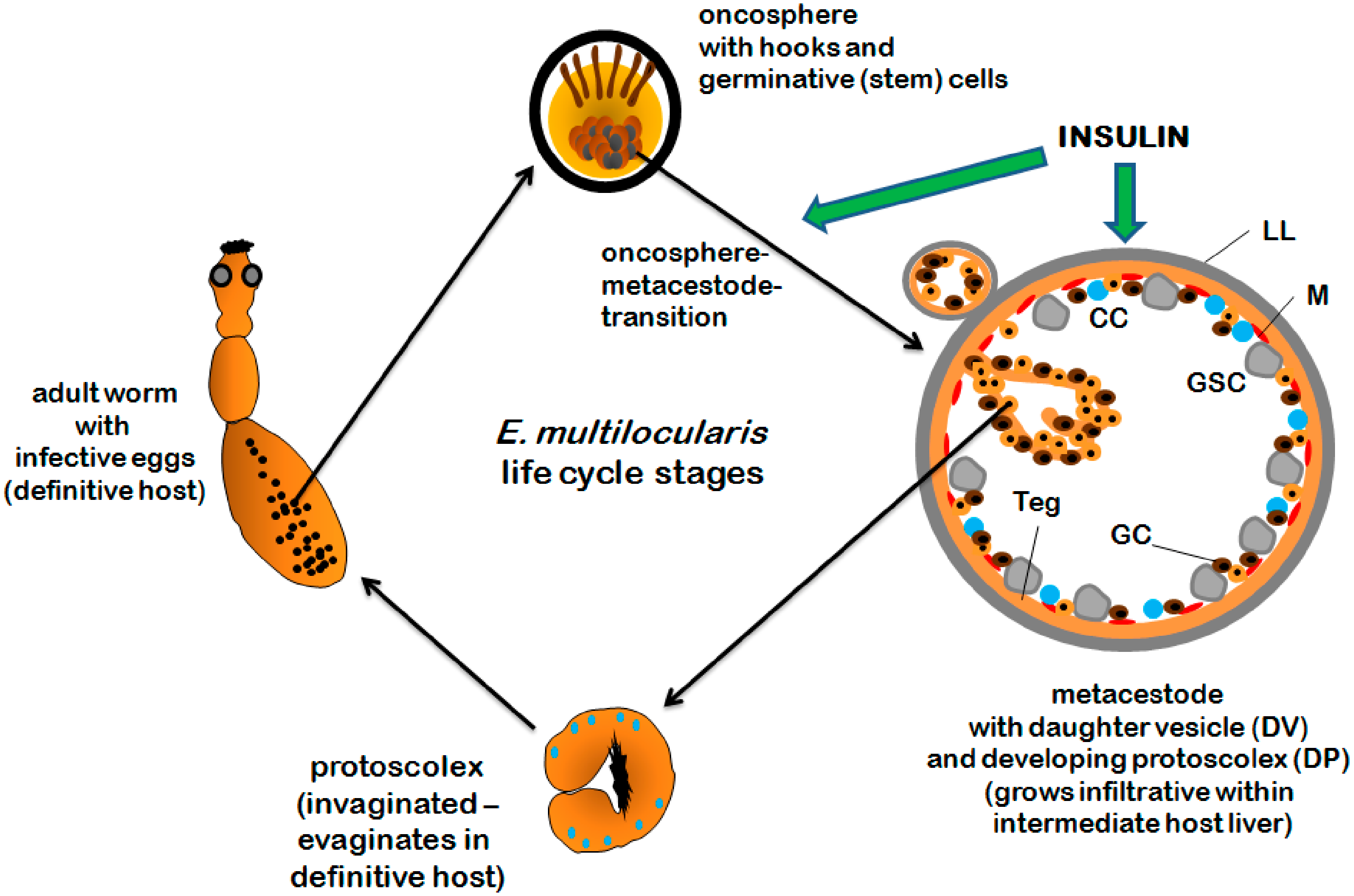

Additional file 1: The *Echinococcus multilocularis* life cycle and proposed actions of host insulin. The *E. multilocularis* life cycle including the developmental stages 'adult worm', 'oncosphere', 'metacestode' and 'protoscolex' is schematically depicted. Proposed positive actions of host insulin on the oncosphere-metacestode-transition and metacestode physiology are indicated by green arrows. Abbreviations are: LL, laminated layer; M, muscle cell; GSC, glycogen storage cell; GC, germinal (stem) cell; Teg, tegument; CC, calcium corpuscle.
