# Supplementary material for: Host insulin stimulates Echinococcus multilocularis insulin signalling pathways and larval development
Source: BMC Biol. 2014 Jan 27;12:5. doi: 10.1186/1741-7007-12-5 (PMC3923246; doi:10.1186/1741-7007-12-5)
Supplement: Additional file 2 — Amino acid sequence comparisons between Echinococcus and human insulin receptors. Pileups of LBDs and TKDs of insulin receptors from Echinococcus and human origin. [file 1741-7007-12-5-S2.pdf]

Additional File 2

A)

```
EmIR1 LBD     SCTVIEGNLLIVMTRLP-----VNASIPNLREITGFLVIYDLMGLDGLATLFPNLTVIR------------GRSLISNFA 63
EmIR2  LBD    NCTVINGNLIISGLSNT-QCIQNGLSLPRLFQIFGSLIVEHSSCSGDLSHFLPNLIAIHGILPFSAQIPIEGKVTIPPYS 79
HsIGF1R LBD   NCTVIEGYLHILLISKA--EDYRSYRFPKLTVITEYLLLFRVAGLESLGDLFPNLTVIR------------GWKLFYNYA 66
HsINSR LBD    NCSVIEGHLQILLMFKTRPEDFRDLSFPKLIMITDYLLLFRVYGLESLKDLFPNLTVIR------------GSRLFFNYA 68

EmIR1 LBD     LVIRSTS-LKTIGLPSLRVIQRGGVRVDLNTNLCYVRTVNWSYILGDQTAAA----APVRLLTNRLICPDTCQPECAAST 138
EmIR2  LBD    LLIHHTA-LSGIGLRCLRVLGRLGVLLIDNPQMCYTDTIGWHLLTPSAYPSKPISNDSVASRNGGGVFGQDLLRKEGLFD 158
HsIGF1R LBD   LVIFEMTNLKDIGLYNLRNITRGAIRIEKNADLCYLSTVDWSLILDAVSN-------------NYIVGNKPP-KECGDLC 132
HsINSR LBD    LVIFEMVHLKELGLYNLMNITRGSVRIEKNNELCYLATIDWSRILDSVED-------------NYIVLNKDDNEECGDIC 135

EmIR1 LBD     PEAISLTELEGTSREKDAKLGHCWSLSYCQSICSANCTSRGIACRMDNTQLCCHKECLAGCYGP-GPEACVACKGALHRG 217
EmIR2  LBD    LCANACPSGCEWVTVNNLPRSFCWSRDHCQHVCAAKCRDASLSCSIHNISECCHANCLGGCTGP-TAGDCLICRDFHHND 237
HsIGF1R LBD   PGTMEEKPMCEKTTINNEYNYRCWTTNRCQKMCPSTCGKRACTE----NNECCHPECLGSCSAPDNDTACVACRHYYYAG 208
HsINSR LBD    PGTAKGKTNCPATVINGQFVERCWTHSHCQKVCPTICKSHGCTA----EGLCCHSECLGNCSQPDDPTKCVACRNFYLDG 211
                                                                                       ***********

EmIR1 LBD     VCVSQCPPSAYLFHGRRCVTAAECFNMSSTYRLPHVAGSSGSSSIGFAASVIVNTTAAVATTAIRQFAIHQGRCVPDCPS 297
EmIR2  LBD    MCLSSCPDGLYELYGRYCVTRNACLSKPIPRDLFSWVFQRSRS-------------------HPSGFSLQGRECVPVCDR 298
HsIGF1R LBD   VCVPACPPNTYRFEGWRCVDRDFCANILS-AESSDSEG----------------------------FVIHDGECMQECPS 259
HsINSR LBD    RCVETCPPPYYHFQDWRCVNFSFCQDLHHKCKNSRRQGC-------------------------HQYVIHNNKCIPECPS 266
              **************************************************************************

EmIR1 LBD     GHQRD-EVSGQCVPCGDECPRIR---CHHMLISSLKSLSKLKDCFSVT-DLYISIHEGDTVLIQQQFDEAFSSLREVES- 371
EmIR2  LBD    SFRRS--PSGECRPCGVDCEEVKR-DCGDIVIYQQADLASVKDCLSAR-SVLISIRAGQD-DLSTSLAEAFSSLRVIYRS 373
HsIGF1R LBD   GFIRNGSQSMYCIPCEGPCPKVCEEEKKTKTIDSVTSAQMLQGCTIFKGNLLINIRRGN--NIASELENFMGLIEVVTGY 337
HsINSR LBD    GYTMN-SSNLLCTPCLGPCPKVCHLLEGEKTIDSVTSAQELRGCTVINGSLIINIRGGN--NLAAELEANLGLIEEISGY 343

EmIR1 LBD     IKVVRATALTSLAFLRHVKRINTIPNSSPN------------VTVIEIRGNDNLLELWPPPTRNQTGGLQVVSEGLVHFI 439
EmIR2  LBD    LRVIRSDALLSLSFLHHLRTIHGTEPIVKDGLVTPVELSHSGITALEITWNGNLEDLWLLPPGSPK---LEILSGSVEFS 450
HsIGF1R LBD   VKIRHSHALVSLSFLKNLRLILGEEQLEGN------------YSFYVLD-NQNLQQLWDWDHRNLT-----IKAGKMYFA 399
HsINSR LBD    LKIRRSYALVSLSFFRKLRLIRGETLEIGN------------YSFYALD-NQNLRQLWDWSKHNLT-----ITQGKLFFH 405

EmIR1 LBD     LNRYLCPKKITDLVRTGALTLPGGRNFRIEEL 471
EmIR2  LBD    VNNRLCPDKIQSFLSN---------------- 466
HsIGF1R LBD   FNPKLCVSEIYRMEEVT--------------- 416
HsINSR LBD    YNPKLCLSEIHKMEEVS--------------- 422
```

Additional File 2: (A) Amino acid sequence comparison between the ligand binding domains (LBD) of *Echinococcus* and human insulin receptor tyrosine kinases. Displayed is a sequence pileup of the LBDs of *E. multilocularis* EmIR1 (AJ458426) and EmIR2 (HG326255; this study) as well as the human insulin receptor (HsINSR; P06213) and the insulin-like growth factor receptor (HsIGF1R; P08069). Amino acid residues that are conserved in at least three of the sequences are shown in white on black background. Biochemically related amino acid residues are marked by a grey background. The specificity determining region for the binding of human insulin-like peptides to the receptors is marked by asterisks below the pileup.

B)

```
                    # # ###
EmIR1 TKD    LNFRHPLGRGNFGMVYRGFVKSLR-------TPAHCFYTEPHNIP----------------------------------- 38
EmIR2 TKD    YNVEDTLGQGSFGLVCRGRLTCLTTPAAEYLHLATTFANGDSASPGGGGGIRPSSTHSALKSKKWLTSILPKRLRRGSVA 80
HsIGF1R TKD  ITMSRELGQGSFGMVYEG--------------VAKGVVKDEPETR----------------------------------- 31
HsINSR TKD   ITLLRELGQGSFGMVYEG--------------NARDIIKGEAETR----------------------------------- 31

                     ## #                #
EmIR1 TKD    --------AAIKTLSSACTVFDRRDFITEACYMKQFQSFHIVRLFGIVSKCSPSSAVPAAARTFLSGGSGSGSGDGGGGF 110
EmIR2 TKD    SGDAQGMDVAVKILSPGSTYEDVREFLGEASHMKQFNCNHIVRLLGIVSKQV---------------------------- 132
HsIGF1R TKD  --------VAIKTVNEAASMRERIEFLNEASVMKEFNCHHVVRLLGVVSQ------------------------------ 73
HsINSR TKD   --------VAVKTVNESASLRERIEFLNEASVMKGFTCHHVVRLLGVVSK------------------------------ 73

EmIR1 TKD    SSAQTKFRFSLFRLFGGGGFWRRHRPKRQAVPVPIKKKLFSLSAEEAMTGNTGNGGSLTRTARTPSNEGFNTSRGPDGSN 190
EmIR2 TKD    -------------------------------------------------------------------------------- 132
HsIGF1R TKD  -------------------------------------------------------------------------------- 73
HsINSR TKD   -------------------------------------------------------------------------------- 73

                                                                                         # #
EmIR1 TKD    GTTDASPTKRVWTGSGRLGRGSFSRLLMKNQKDFRSTLTTQDETGVATVQRCSSSDSIRPFSQYGLFVVMELMESGDLAS 270
EmIR2 TKD    ------------------------------------------------------------LFRRQPIVVMELMQHGDLAT 152
HsIGF1R TKD  --------------------------------------------------------------GQPTLVIMELMTRGDLKS 91
HsINSR TKD   --------------------------------------------------------------GQPTLVVMELMAHGDLKS 91

                                             ##         ##### ##         #  ###         #
EmIR1 TKD    YLRKLG---DSGIGFVKP--AQAYLWAVQIADGMAYLERKKYVHRDLAARNCLVDGRGVVKVGDFGLCRDIYERNYYHKV 345
EmIR2 TKD    YLRHRMAQEDYSQGSVSP--EYAIKWAAEVADGMAYLEYKGFVHRDLAARNCLVGVGLTVKIGDFGLTRDVSGHLYYRKE 230
HsIGF1R TKD  YLRSLRPEMENNPVLAPPSLSKMIQMAGEIADGMAYLNANKFVHRDLAARNCMVAEDFTVKIGDFGMTRDIYETDYYRKG 171
HsINSR TKD   YLRSLRPEAENNPGRPPPTLQEMIQMAAEIADGMAYLNAKKFVHRDLAARNCMVAHDFTVKIGDFGMTRDIYETDYYRKG 171

                  #  #   #          ####  #    #       #                        #
EmIR1 TKD    GAGKLPVRWMAPESLQSAYFTSRSDVWSFGVVLWEIATMACLPYQGMSHNEVISYVLDGNTLVSGGAPINCPPLLQSVML 425
EmIR2 TKD    GRARLPVRWMAPEALNEAYFTFKSDVWSYGVVLWEIATFAALPFSGLSHEEVIALVVNGGHLGKQGWPPKFPDILLDVMQ 310
HsIGF1R TKD  GKGLLPVRWMSPESLKDGVFTTYSDVWSFGVVLWEIATLAEQPYQGLSNEQVLRFVMEGGLLDK---PDNCPDMLFELMR 248
HsINSR TKD   GKGLLPVRWMAPESLKDGVFTTSSDMWSFGVVLWEITSLAEQPYQGLSNEQVLKFVMDGGYLDQ---PDNCPERVTDLMR 248

              ##      ## #
EmIR1 TKD    YCWSYRPAQRPTFLHLLYLL 445
EmIR2 TKD    ACWHSDPECRPSFGTIISML 330
HsIGF1R TKD  MCWQYNPKMRPSFLEIISSI 268
HsINSR TKD   MCWQFNPKMRPTFLEIVNLL 268
```

Additional File 2: (B) Amino acid sequence comparison between the tyrosine kinase domains (TKD) of *Echinococcus* and human insulin receptor tyrosine kinases. Displayed is a sequence pileup of the TKDs of *E. multilocularis* EmIR1 (AJ458426) and EmIR2 (HG326255; this study) as well as the human insulin receptor (HsINSR; P06213) and the insulin-like growth factor receptor (HsIGF1R; P08069). Amino acid residues that are conserved in at least three of the sequences are shown in white on black background. Biochemically related amino acid residues are marked by a grey background. Residues that are highly conserved in eukaryotic tyrosine kinases are indicated by number signs (#) above the alignment.
