# Supplementary material for: Host insulin stimulates Echinococcus multilocularis insulin signalling pathways and larval development
Source: BMC Biol. 2014 Jan 27;12:5. doi: 10.1186/1741-7007-12-5 (PMC3923246; doi:10.1186/1741-7007-12-5)
Supplement: Additional file 3 — Gene expression profiles of Echinococcus insulin signalling components during the life cycle. Diagram displaying transcriptome data concerning the expression of emir1, emir2, emilp1, and emilp2 in larval and adult stages. [file 1741-7007-12-5-S3.pdf]

Additional file 3

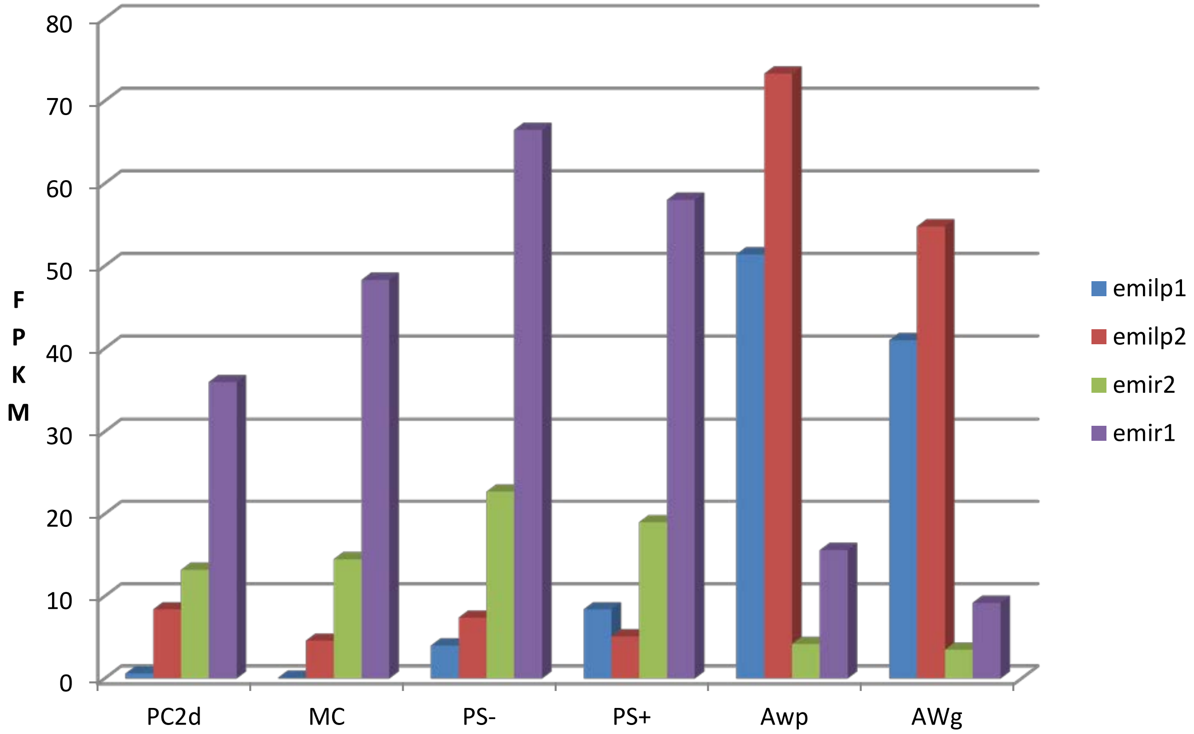

Additional file 3: Gene expression profiles of *Echinococcus* insulin signalling components during the life cycle. Illumina transcriptome sequencing has been carried out for some life cycle stages as support for gene annotation during the *E. multilocularis* whole genome project (Tsai et al., 2013). Shown are FPKM (fragments per kilobase of exon per million fragments mapped) values for *emilp1*, *emilp2*, *emir1*, and *emir2* (as indicated to the right) for primary cells (2 days of cultivation; PC2d), metacestode vesicles (MC), dormant protoscoleces (PS-), pepsin/low pH-activated protoscoleces (PS+), pre-gravid adult worms (Awp), and gravid adult worms (AWg). Please note that Illumina sequencing has been performed only once for each of the samples.

Ref.: Tsai et al. (2013) **The genomes of four tapeworm species reveal adaptations to parasitism**. Nature 496, 57-63.
