# Supplementary material for: Host insulin stimulates Echinococcus multilocularis insulin signalling pathways and larval development
Source: BMC Biol. 2014 Jan 27;12:5. doi: 10.1186/1741-7007-12-5 (PMC3923246; doi:10.1186/1741-7007-12-5)
Supplement: Additional file 4 — Testing the anti-EmIR1 and anti-EmIR2 antisera. File showing Western blot and immunoprecipitation analyses concerning EmIR1 and EmIR2 using the generated antisera. [file 1741-7007-12-5-S4.pdf]

Additional file 4

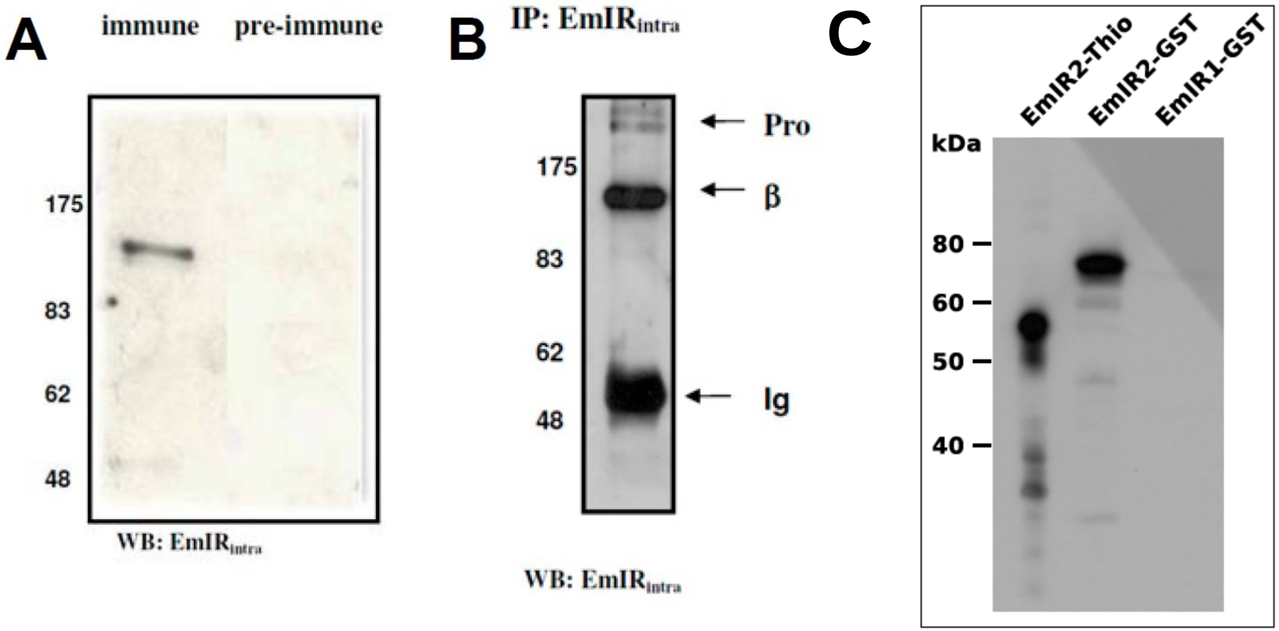

Additional file 4: Testing the anti-EmIR1 and anti-EmIR2 antisera. A) Western blot on whole *E. multilocularis* metacestode lysate with anti-EmIR1 immune serum (left) and pre-immune serum (right). Note that only one band of ~150 kDa is specifically recognized by the antiserum. **B)** Immunoprecipitation of EmIR1 from metacestode lysate. Whole protein from *in vitro* cultivated metacestode vescicles was subjected to immunoprecipitation using the anti-EmIR1 antiserum. Precipitated proteins were separated on a 12.5% PAA gel and transferred to nitrocellulose. Detection was performed with the anti-EmIR1 antiserum. Please note the prominent band at ~150 kDa (β-subunit) and several less intense bands ~190 kDa (pro-form). Ig indicates the antibody band. **C)** Western blot using the anti-EmIR2 antiserum against recombinantly expressed proteins. Fusion proteins of the intracellular portions of EmIR2 and EmIR1 to Thioredoxin and Glutathion S Transferase (GST) were generated as indicated and recombinantly expressed in *E. coli*. The anti-EmIR2 antiserum was then used in Western blot experiments to detect the proteins. Please note that the antiserum strongly recognized EmIR2 fusion proteins, but did not cross-interact with EmIR1 (equal amounts of recombinant protein were loaded).
