# Supplementary material for: Host insulin stimulates Echinococcus multilocularis insulin signalling pathways and larval development
Source: BMC Biol. 2014 Jan 27;12:5. doi: 10.1186/1741-7007-12-5 (PMC3923246; doi:10.1186/1741-7007-12-5)
Supplement: Additional file 5 — In silico analyses. Figure showing in silico models for the binding of HNMPA(AM)3 to the TKD of EmIR1. [file 1741-7007-12-5-S5.pdf]

Additional file 5

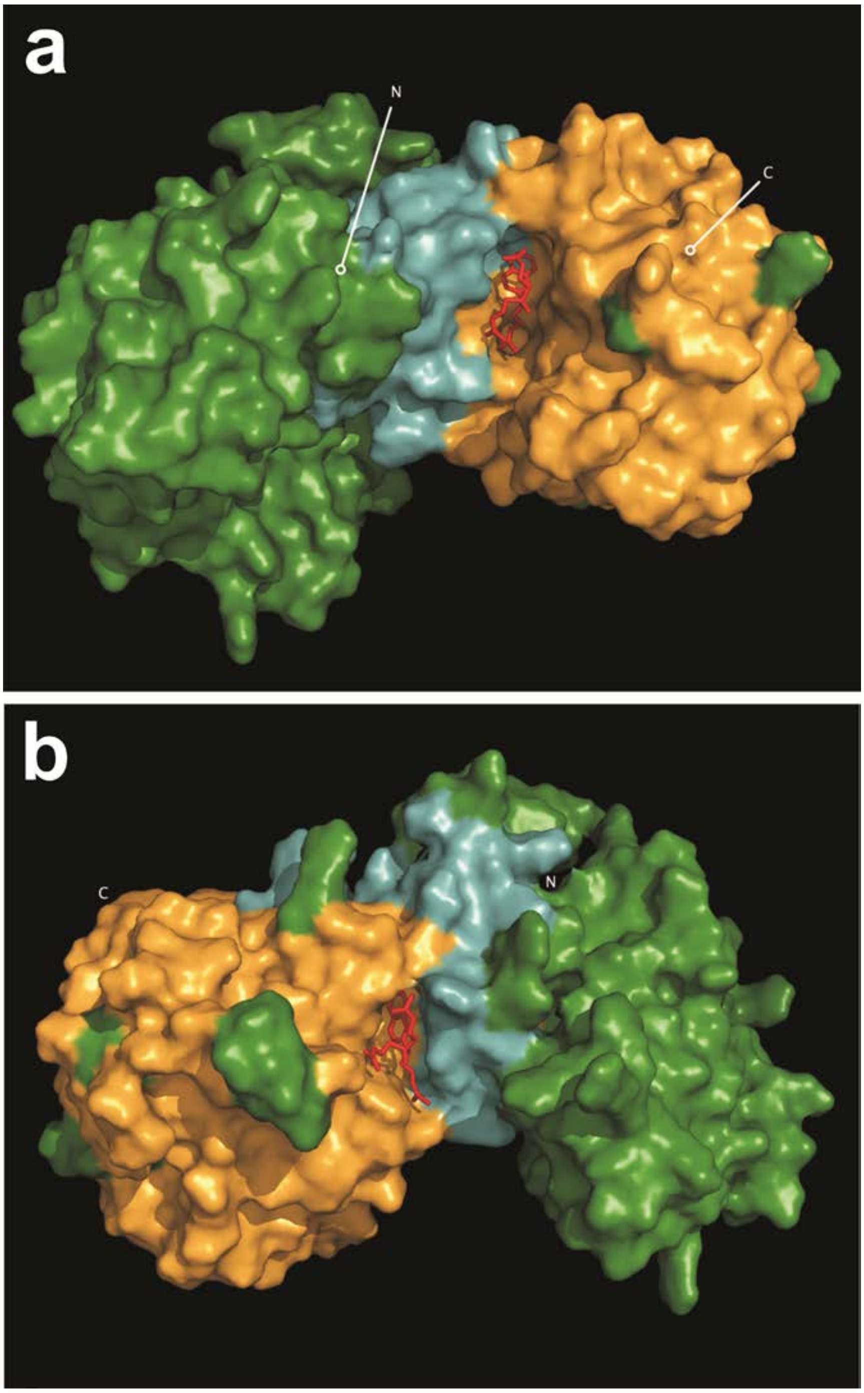

Additional file 5: *In silico* model for HNMPA(AM)$_3$ binding to EmIR1. Results of the CDD prediction superimposed to the predicted HNMPA(AM)3 docking sites of the EmIR1 TKD. N- and C-terminal parts of the protein are depicted in white. The two predicted ATP binding domains are shown in blue and orange. The first and second images (A,B) show the compound's close proximity to these sites and are thus the most likely positions and conformations for competitive inhibition in terms of binding affinity.

Results: The complex of the EmIR1 TKD with HNMPA(AM)$_3$ was built on basis of the SWISS-MODEL prediction for the receptor. For EmIR1 there are two distinct ATP-binding sites according to the Conserved Domain Database (CDD) (Marchler-Bauer et al.,2011). The first is located at amino acids 1 to 293 of the full sequence, while the second is found at amino acids 140 to 445 respectively. The Vina search spaces were defined using this information. Docking analysis reveals two possible binding sites for the HNMPA(AM)$_3$ inhibitor as depicted in Additional file 4. Shown are the most likely solutions for both sites regarding the binding affinities calculated by AutoDock Vina (site a: -7.2 kcal/mol, site b: -7.0 kcal/mol). Based on the information from the CDD prediction, we believe the sites to be relevant for ATP binding. Calculated binding affinities of HNMPA(AM)3 to the human insulin receptor TKD using comparable modeling methods were -4.6 kcal/mol and -6.6 kcal/mol for binding sites a and b, respectively (data not shown).

Methods: Protein structure prediction for EmIR1 was performed using the SWISS-MODEL homology-modelling server (Kiefer et al., 2009). For additional prediction and alternative model generation the MODELLER software package (Eswar et al., 2007) was run locally. Further analysis is based on the structural information obtained from comparable proteins found by SWISS-MODEL and MODELLER. The EmIR1 - HNMPA(AM)$_3$ model was built according to the SWISS-MODEL predicted structures of the receptor. The docking run of the SWISS-MODEL structure was prepared using the PDB2PQR web service (Dolinsky et al., 2007). The chemical structure of the HNMPA(AM)$_3$ inhibitor provided by the supplier (Enzo Life Sciences) was translated to spatial Brookhaven PDB file format using the Online SMILES translator [http://cactus.nci.nih.gov/translate). Additional docking preparation was performed utilizing the two protein sequence BLAST at NCBI and MGLTools AutoDockTools [http://mgltools.scripps.edu). All necessary edits to structural files were made using PyMOL (The PyMOL Molecular Graphics System, Version 1.3.0.0 Schrödinger, LLC). Model evaluation was done using PROCHECK (Lakowski et al., 1993), MODELLER and SWISS-MODEL (Benkert et al., 2008) for stereochemical quality checks and calculation of molpdf, DOPE, GA341 and QMEAN-Z-scores, respectively.

The TKD model provided by SWISS-MODEL covers the complete 445 residue sequence close to the C-terminal part of EmIR1. The QMEAN Z-Score of -4.0 is not optimal but an improvement over full

sequence solutions. Careful inspection of the QMEAN scoring terms show again very good scores in C-beta interaction energy (-1.66), while all-atom pairwise energy (-2.61), secondary structure (-2.24) and solvent accessibility agreements (-2.58) still scored good. Solvation (-4.66) and torsion angle energies (-4.60) give room to improve the model. All structures were visualized using the PyMOL software package.

References:

Benkert P, et al.: **QMEAN: A comprehensive scoring function for model quality assessment.** *Proteins* 2008, **71:**261-277.

Dolinsky TJ, et al.: **PDB2PQR: Expanding and upgrading automated preparation of biomolecular structures for molecular simulations.** *Nucleic Acids Res* 2007, **35:**W522-525.

Eswar N, et al.: (2006) **Comparative protein structure modeling using MODELLER.** *Curr Protoc Protein Sci* 2007, Chapter 2: Unit 2.9.

Garrett TPJ, et al.: **Crystal structure of the first three domains of the type-1 insulin-like growth factor receptor.** *Nature* 1998, **394:**395–399.

Kiefer F, et al.: **The SWISS-MODEL repository and associated resources.** *Nucleic Acids Res* 2009, **37:**D387-D392.

Laskowski RA, et al.: **PROCHECK - a program to check the stereochemical quality of protein structures**. *J App Cryst* 1993, **26:**283-291.

Marchler-Bauer A, et al.: **CDD: a conserved domain database for the functional annotation of proteins.** *Nucleic Acids Res* 2011, **39:**225-229.
