# Supplementary material for: Host insulin stimulates Echinococcus multilocularis insulin signalling pathways and larval development
Source: BMC Biol. 2014 Jan 27;12:5. doi: 10.1186/1741-7007-12-5 (PMC3923246; doi:10.1186/1741-7007-12-5)
Supplement: Additional file 6 — Components of the E. multilocularis insulin signalling pathways. Table with E. multilocularis genes predicted to be involved in insulin signalling pathways according to the E. multilocularis genome sequencing project. [file 1741-7007-12-5-S6.pdf]

Additional file 6

Product	Systematic name	Location	Homology
Em4E-BP	EmuJ_000044600	contig 60709: 459698-463653	eukaryotic translation initiation factor 4E binding protein
EmAkt	EmuJ_000979200	scaffold 7780: 4083862-4092994	akt-like serine/threonine protein kinase
EmFKBP12	EmuJ_000787900	scaffold 7768: 5264722-5284913	FKBP12 rapamycin complex associated protein
EmGSK3	EmuJ_000980300	scaffold 7780: 4164112-4186701	glycogen synthase kinase 3
EmIF-4E	EmuJ_000809800	scaffold 7768: 6884517-6885510	eukaryotic translation initiation factor 4E
EmIRS	EmuJ_000848300	scaffold 7768: 10045618-10058030	insulin receptor substrate
EmPI3K	EmuJ_000526100	scaffold 7636: 1191161-1207682	phosphatidylinositol 3-kinase, catalytic, alpha
EmPDK1	EmuJ_000723900	scaffold 7765: 22476-24029	phosphoinositide-dependent protein kinase 1
EmPKC	EmuJ_000455600	scaffold 7614: 3119007-3125424	protein kinase C iota type
RmRps6	EmuJ_000877900	scaffold 7768: 12109605-12110518	ribosomal protein S6 kinase
EmTOR	EmuJ_000787900	scaffold 7768: 5264722-5284913	target of rapamycin

Additional file 6: Components of the *E. multilocularis* insulin signaling pathway as predicted by the genome project. The table lists *E. multilocularis* orthologs of genes involved in insulin signaling pathways in other organisms. Indicated are the names of the encoded proteins, the systematic designation of the gene, the location on contigs and supercontigs (scaffolds) of the *E. multilocularis* genome (Tsai et al., 2013) and the gene products. The respective information can be found in (http://www.genedb.org/Homepage/Emultilocularis).

Ref.: Tsai et al. (2013) **The genomes of four tapeworm species reveal adaptations to parasitism**. Nature 496, 57-63.
