# Supplementary material for: Host insulin stimulates Echinococcus multilocularis insulin signalling pathways and larval development
Source: BMC Biol. 2014 Jan 27;12:5. doi: 10.1186/1741-7007-12-5 (PMC3923246; doi:10.1186/1741-7007-12-5)
Supplement: Additional file 7 — Amino acid sequence comparison of PKB components from different organisms. File showing sequence alignment of the E. multilocularis PKB-homolog EmAkt with PKBs from human and Drosophila origin. [file 1741-7007-12-5-S7.pdf]

Additional file 7

```
EmAkt   MQVDESLNCSTPMGTLTPTSAPMAVDPLSSFVQFQSASMSQIPCNTPQFSNPSTVSSLVPGAGTPCAGLSQEHFVPHAPV 80
DmAKT1  ------MNYLPFVLQRRSTVVASAPAPGSASRIPESPTTTGSNIINIIYS-QSTHPNSSPTSGSAEKFSWQQSWPSRTSA 73
HsAKT3  -------------------------------------------------------------------------------- 1

EmAkt   SMPHLTGGLPGSTDLLGHQQALAINPTSLMQYFRIRTLPLTRKVIREGWLMKRGEHIKTWRRRYFILREDGTFYGYKNIP 160
DmAKT1  APTHDSGTMSINTTFDLSSPSVTSG----------HALTEQTQVVKEGWLMKRGEHIKNWRQRYFVLHSDGRLMGYRSKP 143
HsAKT3  --------------------------------------MSDVTIVKEGWVQKRGEYIKNWRPRYFLLKTDGSFIGYKEKP 42

EmAkt   RDNLEQP----LNNFTVRDCQIICLNKPKPYTILMRGLQWTTVVERLFFVEHEVERDEWISAIQMVANRLRSENEAPTSV 236
DmAKT1  ADSASTPSDFLLNNFTVRGCQIMTVDRPKPFTFIIRGLQWTTVIERTFAVESELERQQWTEAIRNVSSRLIDVGEVAMTP 223
HsAKT3  QD-VDLP--YPLNNFSVAKCQLMKTERPKPNTFIIRCLQWTTVIERTFHVDTPEEREEWTEAIQAVADRLQRQEEERMNC 119

EmAkt   FKVD---------FAEDVVIDFPQRP--------PKRYSTDDFELLKVLGKGTFGKVVLCKEKESGCFYAMKILKKTVLI 299
DmAKT1  SEQTDMTDVDMATIAEDELSEQFSVQGTTCNSSGVKKVTLENFEFLKVLGKGTFGKVILCREKATAKLYAIKILKKEVII 303
HsAKT3  SPTSQIDN-----IGEEEMDASTTHH---------KRKTMNDFDYLKLLGKGTFGKVILVREKASGKYYAMKILKKEVII 185

EmAkt   EKEEVGHTQTEHRVLQLNHHPFMTQLKYSFTTRDHIFFVMEYCNGGELFYHLSREHVFSESRTQFYAAEITSALGYLHSQ 379
DmAKT1  QKDEVAHTLTESRVLKSTNHPFLISLKYSFQTNDRLCFVMQYVNGGELFWHLSHERIFTEDRTRFYGAEIISALGYLHSQ 383
HsAKT3  AKDEVAHTLTESRVLKNTRHPFLTSLKYSFQTKDRLCFVMEYVNGGELFFHLSRERVFSEDRTRFYGAEIVSALDYLHSG 265

EmAkt   NIVYRDLKLENLLLDKDGHIKITDFGLCKEDIGFGSTTKTFCGTPEYLAPELLLDNDYGLSVDWWSLGVVMYEMMCGRLP 459
DmAKT1  GIIYRDLKLENLLLDKDGHIKVADFGLCKEDITYGRTTKTFCGTPEYLAPEVLDDNDYGQAVDWWGTGVVMYEMICGRLP 463
HsAKT3  KIVYRDLKLENLMLDKDGHIKITDFGLCKEGITDAATMKTFCGTPEYLAPEVLEDNDYGRAVDWWGLGVVMYEMMCGRLP 345

EmAkt   FYSNEHEILFELILQESVKVPDNLSPVARDILIRLLMKDPAERLGGGKADAIEVMVHPFFESISWDKLIRKDIIPPWKPD 539
DmAKT1  FYNRDHDVLFTLILVEEVKFPRNITDEAKNLLAGLLAKDPKKRLGGGKDDVKEIQAHPFFASINWTDLVLKKIPPPFKPQ 543
HsAKT3  FYNQDHEKLFELILMEDIKFPRTLSSDAKSLLSGLLIKDPNKRLGGGPDDAKEIMRHSFFSGVNWQDVYDKKLVPPFKPQ 425

EmAkt   VNGDMDTKYIPEEFQRENVAVTPPE--KSVASAIMAADRVSVVKVFSASETMALYLKAPFPTDSPTLSSAKAQSLTVYIR 617
DmAKT1  VTSDTDTRYFDKEFTGESVELTPPD--PTGPLGSIAEE--PLFPQFSYQGDMASTLG----TSSHISTSTSLASMQ---- 611
HsAKT3  VTSETDTRYFDEEFTAQTITITPPEKYDEDGMDCMDNERRPHFPQFSYSASGRE-------------------------- 479

EmAkt   GTFGGSFPVDVITVRRIQPNSPSLRRIFGHAYFSPRGQLFDSCLRVSFVEIMVISHCVSLCLFISLSIPATWHFSCTIAD 697
DmAKT1  -------------------------------------------------------------------------------- 611
HsAKT3  -------------------------------------------------------------------------------- 479

EmAkt   AVVRPLLSSLPLWDV 712
DmAKT1  --------------- 611
HsAKT3  --------------- 479
```

Additional file 7: Amino acid sequence comparison of protein kinase B (Akt) proteins from different organisms. Aligned are sequences of *E. multilocularis* (EmAkt), human (HsAkt3; accession number Q9Y243) and *Drosophila* (DmAkt1; Q24293) origin. Amino acid residues identical in all three proteins are shown in white on black background. Amino acid sequence identity and similarity values between EmAkt and HsAKT3 were 56% and 70%, respectively, those between EmAkt and DmAKT1 were 55%/70%. The EmAkt cDNA sequence has been submitted to the EMBL database and be assigned accession number HF934007.
