# Supplementary material for: Host insulin stimulates Echinococcus multilocularis insulin signalling pathways and larval development
Source: BMC Biol. 2014 Jan 27;12:5. doi: 10.1186/1741-7007-12-5 (PMC3923246; doi:10.1186/1741-7007-12-5)
Supplement: Additional file 8 — Amino acid sequence comparison of 4E-BP orthologs from different origin. File showing sequence alignment of the E. multilocularis 4E-BP ortholog with those of human and insect origin. [file 1741-7007-12-5-S8.pdf]

Additional file 8

```
Em4EBP    MASNRGPDGIP------FRRLKVTDPSQIP-NDYSTTPGGSIFSTTPGGTRIFYDRDTMLMCKNSPIARSPPTDMVCRIT 73
Hs4EBP1   MSGGSSCSQTPSRAIPATRRVVLGDGVQLPPGDYSTTPGGTLFSTTPGGTRIIYDRKFLMECRNSPVTKTPPRDLPTIPG 80
                                                    *       *                   *    *

Em4EBP    CPATCAGECRLPAATAQKPAKNTRQEHASVQKSDEGPFDIDL 115
Hs4EBP1   VTSPSSDEP--PMEASQSHLRNSPEDKRAG--GEESQFEMDI 118
```

Additional file 8: Amino acid sequence comparison of EIF4E binding proteins (4EBPs) from *E. multilocularis* and human origin. Aligned are sequences of *E. multilocularis* (Em4EBP) and *Homo spaiens* human (Hs4EBP1; accession number Q13541). Amino acid residues identical in both proteins are shown in white on black background. Residues with similar function are printed in black on grey background. Amino acid sequence identity and similarity values between Em4EBP and Hs4EBP1 were 40% and 64%. Asterisks indicate phosphorylation sites in human 4E-BP. The Em4EBP cDNA sequence has been submitted to the EMBL database and been assigned accession number HF934006.
